# Supplementary material for: Unpacking lithic assemblage variability in the Early Upper Palaeolithic: A multivariate approach to the structure of the Iberian Aurignacian
Source: PLoS One. 2026 Mar 27;21(3):e0345202. doi: 10.1371/journal.pone.0345202 (PMC13028375; doi:10.1371/journal.pone.0345202)
Supplement: S1 Table — Processed binary data of techno-typological attributes. (ZIP) [file pone.0345202.s001.zip › S1_Table_references.docx]

**References for S1 Table**

Almeida, F., 2001. Cores, tools, or both? Methodological contribution for the study of carinated lithic elements: the Portuguese case. In: M.A. Hays and P.T. Thacker, (Eds.). *Questioning the Answers: Re-solving Fundamental Problems of the Early Upper Paleolithic*. Oxford: British Archaeological Reports, pp. 91–98.

Arrizabalaga, A., 2000a. Los tecnocomplejos líticos del yacimiento arqueológico de Labeko Koba (Arrasate, País Vasco). In: *Labeko Koba (País Vasco): Hienas y humanos en los albores del Paleolítico superior*. San Sebastián–Donostia: Sociedad de Ciencias Aranzadi, pp. 193–343.

Aubry, T., Dimuccio, L.A., Barbosa, A.F., Luís, L., Santos, A.T., Silvestre, M., Thomsen, K.J., Rades, E., Autzen, M., and Murray, A.S., 2020. Timing of the Middle-to-Upper Palaeolithic transition in the Iberian inland (Cardina-Salto do Boi, Côa Valley, Portugal). *Quaternary Research*, 98, 81–101. <https://doi.org/10.1017/qua.2020.43>.

Barton, R.N.E. and Jennings, R.P., 2012. The lithic artefact assemblages of Gorham’s Cave. In: *Neanderthals in Context: a report of the 1995–1998 excavations at Gorham’s and Vanguard Caves, Gibraltar*. Oxford: Oxford University School of Archaeology, pp. 151–187.

Bernaldo de Quirós, F. and Maíllo-Fernández, J.M., 2009. The Transitional Aurignacian and the Middle-Upper Palaeolithic Transition Model in Cantabrian Iberia. In: M. Camps and P. Chauhan, (Eds.). *Sourcebook of Paleolithic Transitions: Methods, Theories, and Interpretations*. New York, NY: Springer, pp. 341–359.

Bicho, N.F., 2005. The extinction of Neanderthals and the emergence of the Upper Paleolithic in Portugal. *Promontoria*, (5), 173–228.

Cortés Sánchez, M., 2007a. Las industrias líticas del Paleolítico Medio y del Paleolítico Superior. In: M. Cortés Sánchez, (Ed.). *Cueva de Bajondillo (Torremolinos). Secuencia cronocultural paleoambiental del Cuaternario Reciente en la Bahía de Málaga*. Málaga: Servicio de Publicaciones, Centro de Ediciones de la Diputación de Málaga, pp. 171–446.

Haws, J., Cascalheira, J., and Benedetti, M., 2023. Inquiry into modern human distributions [dataset]. https://osf.io/8zrqy/

Maíllo Fernández, J.M., 2003. La transición paleolítico medio-superior en Cantabria: análisis tecnológico de la industria lítica de Cueva Morín. PhD thesis. Universidad Nacional de Educación a Distancia (UNED).

Maíllo-Fernández, J.M. and Bernaldo de Quirós, F., 2010. L’Aurignacien archaïque de la grotte El Castillo (Espagne): caractérisation technologique et typologique. *L’Anthropologie*, 114(1), 1–25. <https://doi.org/10.1016/j.anthro.2010.01.001>.

Martínez-Moreno, J., Mora, R., de la Torre, I., and Benito-Calvo, A., 2012. The role of flakes in the early Upper Palaeolithic 497D assemblage of Cova Gran de Santa Linya (southeastern pre-pyrenees, Spain). In: A. Pastoors and M. Peresani, (Eds.). Mettmann: Neanderthal Museum, pp. 85–104.

Martínez-Moreno, J., Mora Torcal, R., Benito-Calvo, A., Roy Sunyer, M., and Sánchez-Martínez, J., 2019. A bunch of refits: 497D blade knapping assemblage of the Early Upper Paleolithic in Cova Gran (Northeast Iberia). *Archaeological and Anthropological Sciences*, 11(9), 4585–4600. <https://doi.org/10.1007/s12520-018-0726-3>.

Morales, J.I., Cebrià, A., Burguet-Coca, A., Fernández-Marchena, J.L., García-Argudo, G., Rodríguez-Hidalgo, A., Soto, M., Talamo, S., Tejero, J.-M., Vallverdú, J., and Fullola, J.M., 2019. The Middle-to-Upper Paleolithic transition occupations from Cova Foradada (Calafell, NE Iberia). *PLOS ONE*, 14(5), e0215832. <https://doi.org/10.1371/journal.pone.0215832>.

Ortega, D., 2002. Mobilitat i desplaçaments dels grups caçadors-recol.lectors a inicis del paleolític superior a la regió pirinenca oriental. *Cypsela*, (14), 11–26.

Ortega, D., Soler, N., and Maroto, J., 2005. La prodution de lamelles pendant l’Aurignacien archaïque dans la grotte de l’Arbreda: organisation de la production, variabilité des méthodes et des objectifs. In: F. Le Brun-Ricalens, J.-G. Bordes, and F. Bon, (Eds.). *Productions lamellaires attribuées à l’Aurignacien: chaînes opératoires et perspectives technoculturelles*. Luxembourg: Musée National d’Histoire et d’Art, pp. 359–373.

Pereira, T.J., 2011. A new explanatory model for the first Upper Paleolithic occupations in SW Iberia. *Estrat crític*, 5(3), 156–164.

Rios Garaizar, J., de la Peña, P., and San Emeterio, A., 2011. Estudio de las industrias líticas y óseas de la cueva de Aitzbitarte III (Zona de la entrada). In: J. Altuna, K. Mariezkurrena, and J. Rios-Garaizar, (Eds.). *Ocupaciones humanas en la cueva de Aitzbitarte III (Renteria, País Vasco) sector Entrada: 33.000-18.000 BP*. Vitoria: Servicio Central de Publicaciones del Gobierno Vasco, pp. 81–351.

Rios-Garaizar, J., 2011. El nivel IXb de Ekain (Deba, Gipuzkoa, Región Cantábrica): Una ocupación efímera del Auriñaciense Antiguo. *Munibe Antropologia-Arkeologia*, 62, 87–100.

Sala, N., Alcaraz-Castaño, M., Arriolabengoa, M., Martínez-Pillado, V., Pantoja-Pérez, A., Rodríguez-Hidalgo, A., Téllez, E., Cubas, M., Castillo, S., Arnold, L.J., Demuro, M., Duval, M., Arteaga-Brieba, A., Llamazares, J., Ochando, J., Cuenca-Bescós, G., Marín-Arroyo, A.B., Seijo, M.M., Luque, L., Alonso-Llamazares, C., Arlegi, M., Rodríguez-Almagro, M., Calvo-Simal, C., Izquierdo, B., Cuartero, F., Torres-Iglesias, L., Agudo-Pérez, L., Arribas, A., Carrión, J.S., Magri, D., Zhao, J.-X., and Pablos, A., 2024. Nobody’s land? The oldest evidence of early Upper Paleolithic settlements in inland Iberia. *Science Advances*, 10(26), eado3807. <https://doi.org/10.1126/sciadv.ado3807>.

Santamaría Álvarez, D., 2012. La transición del Paleolítico medio al superior en Asturias. El Abrigo de La Viña (La Manzaneda, Oviedo) y la Cueva de El Sidrón (Borines, Piloña). PhD thesis. Universidad de Oviedo.

Verdún-Castelló, E. and Casabó i Bernad, J., 2020. Shellfish consumption in the Early Upper Palaeolithic on the Mediterranean coast of the Iberian Peninsula: The example of Foradada Cave. *Journal of Archaeological Science: Reports*, 29, 102035. <https://doi.org/10.1016/j.jasrep.2019.102035>.

Villaverde, V., Real, C., Roman, D., Albert, R.M., Badal, E., Bel, M.Á., Bergadà, M.M., de Oliveira, P., Eixea, A., Esteban, I., Martínez-Alfaro, Á., Martínez-Varea, C.M., and Pérez-Ripoll, M., 2019. The early Upper Palaeolithic of Cova de les Cendres (Alicante, Spain). *Quaternary International*, 515, 92–124. <https://doi.org/10.1016/j.quaint.2017.11.051>.

Villaverde, V., Sanchis, A., Badal, E., Bel, M.Á., Bergadà, M.M., Eixea, A., Guillem, P.M., Martínez-Alfaro, Á., Martínez-Valle, R., Martínez-Varea, C.M., Real, C., Steier, P., and Wild, E.M., 2021. Cova de les Malladetes (Valencia, Spain): New Insights About the Early Upper Palaeolithic in the Mediterranean Basin of the Iberian Peninsula. *Journal of Paleolithic Archaeology*, 4(1), 5. <https://doi.org/10.1007/s41982-021-00081-w>.

Zilhão, J., 1997. *O Paleolítico Superior da Estremadura portuguesa*. Lisboa: Colibri.

Zilhão, J., Anesin, D., Aubry, T., Badal, E., Cabanes, D., Kehl, M., Klasen, N., Lucena, A., Martín-Lerma, I., Martínez, S., Matias, H., Susini, D., Steier, P., Wild, E.M., Angelucci, D.E., Villaverde, V., and Zapata, J., 2017. Precise dating of the Middle-to-Upper Paleolithic transition in Murcia (Spain) supports late Neandertal persistence in Iberia. *Heliyon*, 3(11), e00435. <https://doi.org/10.1016/j.heliyon.2017.e00435>.
