# Supplementary material for: Unpacking lithic assemblage variability in the Early Upper Palaeolithic: A multivariate approach to the structure of the Iberian Aurignacian
Source: PLoS One. 2026 Mar 27;21(3):e0345202. doi: 10.1371/journal.pone.0345202 (PMC13028375; doi:10.1371/journal.pone.0345202)
Supplement: S2 Table — Processed radiocarbon and luminescence dates are accompanied by calibrated and median ages. These reported ages were used to calculate the median assemblage ages for partial Mantel tests. (ZIP) [file pone.0345202.s002.zip › S2_Table_references.docx]

**References for S2 Table**

Altuna, J., Mariezkurrena, K., and Rios Garaizar, J., eds., 2011. *Ocupaciones humanas en Aitzbitarte III (País Vasco) 33.600-18.400 BP: (Zona de entrada a la cueva)*. Vitoria: Servicio Central de Publicaciones del Gobierno Vasco.

Altuna, Jesús. and Merino, J.M., 1984. *El yacimiento prehistórico de la cueva de Ekain (Deba, Guipuzcoa)*. San Sebastián: Sociedad de Estudios Vascos.

Arrizabalaga, A., 2000. El yacimiento arqueológico de Labeko Koba (Arrasate, País Vasco): Entorno, crónica de las investigaciones, estratigrafía y estructuras: Cronología absoluta. In: *Labeko Koba (País Vasco): Hienas y humanos en los albores del Paleolítico superior*. San Sebastián–Donostia: Sociedad de Ciencias Aranzadi, pp. 15–72.

Aubry, T., Dimuccio, L.A., Barbosa, A.F., Luís, L., Santos, A.T., Silvestre, M., Thomsen, K.J., Rades, E., Autzen, M., and Murray, A.S., 2020. Timing of the Middle-to-Upper Palaeolithic transition in the Iberian inland (Cardina-Salto do Boi, Côa Valley, Portugal). *Quaternary Research*, 98, 81–101. <https://doi.org/10.1017/qua.2020.43>.

Bischoff, J.L., Soler, N., Maroto, J., and Julià, R., 1989. Abrupt Mousterian/Aurignacian boundary at c. 40 ka bp: Accelerator 14C dates from l’Arbreda Cave (Catalunya, Spain). *Journal of Archaeological Science*, 16(6), 563–576. <https://doi.org/10.1016/0305-4403(89)90022-8>.

Cabrera-Valdés, V., Valladas, H., Bernaldo de Quiros, F., and Hoyos Gomez, M., 1996. La transition Paléolithique moyen-Paléolithique supérieur à El Castillo (Cantabrie): nouvelles datation par le carbone. *Comptes Rendus de l’Académie des Sciences - Series III - Sciences de la Vie*, 322, 1093–1098.

Cortés Sánchez, M., 2007. *El Paleolítico Medio y Superior en el sector central de Andalucía (Córdoba y Málaga)*. Madrid: Museo de Altamira Monografias.

Cortés-Sánchez, M., Jiménez-Espejo, F.J., Simón-Vallejo, M.D., Stringer, C., Lozano Francisco, M.C., García-Alix, A., Vera Peláez, J.L., Odriozola, C.P., Riquelme-Cantal, J.A., Parrilla Giráldez, R., Maestro González, A., Ohkouchi, N., and Morales-Muñiz, A., 2019. An early Aurignacian arrival in southwestern Europe. *Nature Ecology & Evolution*, 3(2), 207–212. <https://doi.org/10.1038/s41559-018-0753-6>.

Delibrias, G., Romain, O., and Le Hasif, G., 1987. Datation par la méthode du carbone 14 du remplissage de la grotte de l’Arbreda. *Cypsela*, (6), 133–135.

Fortea Pérez, F.J., 1995. Abrigo de la Viña: informe y primera valoración de las campañas 1991 a 1994. In: *Excavaciones Arqueológicas en Asturias: 1991-94*. Oviedo: Consejería de Cultura, pp. 19–32.

Fortea Pérez, F.J., 1999. Abrigo de la Viña: informe y primera valoración de las campañas de 1995 a 1998. In: *Excavaciones Arqueológicas en Asturias: 1995-98*. Oviedo: Consejería de Cultura, pp. 31–41.

Fortea Pérez, F.J. and Jordá Cerdá, F., 1976. La Cueva de Les Mallaetes y los problemas del Paleolítico Superior del Mediterráneo español. *Zephyrus: Revista de prehistoria y arqueología*, (26), 129–166.

Haws, J.A., 2012. Paleolithic socionatural relationships during MIS 3 and 2 in central Portugal. *Quaternary International*, 264, 61–77. <https://doi.org/10.1016/j.quaint.2011.10.003>.

Haws, J.A., Benedetti, M.M., Talamo, S., Bicho, N., Cascalheira, J., Ellis, M.G., Carvalho, M.M., Friedl, L., Pereira, T., and Zinsious, B.K., 2020. The early Aurignacian dispersal of modern humans into westernmost Eurasia. *Proceedings of the National Academy of Sciences*, 117(41), 25414–25422. <https://doi.org/10.1073/pnas.2016062117>.

Hedges, R.E.M., Housley, R.A., Ramsey, C.B., and Van Klinken, G.J., 1994. Radiocarbon Dates from the Oxford Ams System: Archaeometry Date List 18. *Archaeometry*, 36(2), 337–374. <https://doi.org/10.1111/j.1475-4754.1994.tb00975.x>.

Higham, T.F.G., Bronk Ramsey, C., Cheney, H., Brock, F., and Douka, K., 2012. The radiocarbon chronology of Gorham’s Cave. In: R.N.E. Barton, C.B. Stringer, and C. Finlayson, (Eds.). *Neanderthals in Context: a report of the 1995–1998 excavations at Gorham’s and Vanguard Caves, Gibraltar*. Oxford: Oxford University School of Archaeology, pp. 62–76.

Maíllo-Fernández, J.M., Arteaga, C., Iriarte-Chiapusso, M., Fernández, A., Wood, R., and Bernaldo de Quiros, F., 2014. Cueva Morín (Villanueva de Villaescusa, Cantabria). In: R. Sala Ramos, (Ed.). *Pleistocene and Holocene hunter-gatherers in Iberia and the Gibraltar strait: the current archaeological record*. Burgos: Universidad de Burgos, pp. 72–78.

Maíllo-Fernández, J.M.M., Valladas, H., Cabrera Valdés, V., and de Quirós, F.B., 2001. Nuevas dataciones para el Paleolítico superior de Cueva Morin (Villanueva de Villaescusa, Cantabria). *Espacio Tiempo y Forma. Serie I, Prehistoria y Arqueología*, (14).

Marín-Arroyo, A.B., Rios-Garaizar, J., Straus, L.G., Jones, J.R., Rasilla, M. de la, Morales, M.R.G., Richards, M., Altuna, J., Mariezkurrena, K., and Ocio, D., 2018. Chronological reassessment of the Middle to Upper Paleolithic transition and Early Upper Paleolithic cultures in Cantabrian Spain. *PLOS ONE*, 13(4), e0194708. <https://doi.org/10.1371/journal.pone.0194708>.

Maroto, J., Vaquero, M., Arrizabalaga, Á., Baena, J., Baquedano, E., Jordá Pardo, J.F., Julià, R., Montes, R., Plicht, J. van del, Rasines, P., and Wood, R., 2012. Current Issues in Late Middle Palaeolithic Chronology: the Curious Case of Northern Iberia. *Quaternary International*, (247), 15–25.

Martínez-Moreno, J., Mora, R., and Ignacio de la Torre, 2010. The Middle-to-Upper Palaeolithic transition in Cova Gran (Catalunya, Spain) and the extinction of Neanderthals in the Iberian Peninsula. *Journal of Human Evolution*, 58(3), 211–226. <https://doi.org/10.1016/j.jhevol.2009.09.002>.

Mora, R., Martínez-Moreno, J., Roy Sunyer, M., Benito Calvo, A., Polo-Díaz, A., and Samper Carro, S., 2018. Contextual, technological and chronometric data from Cova Gran: Their contribution to discussion of the Middle-to-Upper Paleolithic transition in northeastern Iberia. *Quaternary International*, 474, 30–43. <https://doi.org/10.1016/j.quaint.2016.05.017>.

Morales, J.I., Cebrià, A., Burguet-Coca, A., Fernández-Marchena, J.L., García-Argudo, G., Rodríguez-Hidalgo, A., Soto, M., Talamo, S., Tejero, J.-M., Vallverdú, J., and Fullola, J.M., 2019. The Middle-to-Upper Paleolithic transition occupations from Cova Foradada (Calafell, NE Iberia). *PLOS ONE*, 14(5), e0215832. <https://doi.org/10.1371/journal.pone.0215832>.

Pettitt, P.B. and Bailey, R.M., 2000. AMS Radiocarbon and Luminescnece Dating of Gorham’s and Vanguard Caves, Gibraltar, and Implications for the Middle to Upper Palaeolithic Transition in Iberia. In: C.B. Stringer, R.N.E. Barton, and J.C. Finlayson, (Eds.). *Neanderthals on the Edge*. Oxford: Oxbow Books, pp. 155–162.

Sala, N., Alcaraz-Castaño, M., Arriolabengoa, M., Martínez-Pillado, V., Pantoja-Pérez, A., Rodríguez-Hidalgo, A., Téllez, E., Cubas, M., Castillo, S., Arnold, L.J., Demuro, M., Duval, M., Arteaga-Brieba, A., Llamazares, J., Ochando, J., Cuenca-Bescós, G., Marín-Arroyo, A.B., Seijo, M.M., Luque, L., Alonso-Llamazares, C., Arlegi, M., Rodríguez-Almagro, M., Calvo-Simal, C., Izquierdo, B., Cuartero, F., Torres-Iglesias, L., Agudo-Pérez, L., Arribas, A., Carrión, J.S., Magri, D., Zhao, J.-X., and Pablos, A., 2024. Nobody’s land? The oldest evidence of early Upper Paleolithic settlements in inland Iberia. *Science Advances*, 10(26), eado3807. <https://doi.org/10.1126/sciadv.ado3807>.

Soler, N. and Maroto, J., 1993. Les nouvelles datations de l’Aurignacien dans la Péninsule Ibérique. In: L. Bánesz and J.K. Kozlowski, (Eds.). *Aurignacien en Europe et au Proche Orient, Actes du XIIe Congrès International des Sciences Préhistoriques et Protohistoriques*. pp. 162–173.

Stuckenrath, R., 1978. Dataciones de Carbono 14. In: J. González Echegaray and L.G. Freeman, (Eds.). *Vida y muerte en Cueva Morín*. Santander: Institución Cultural de Cantabria, pp. 215–216.

Villaverde, V., Real, C., Roman, D., Albert, R.M., Badal, E., Bel, M.Á., Bergadà, M.M., de Oliveira, P., Eixea, A., Esteban, I., Martínez-Alfaro, Á., Martínez-Varea, C.M., and Pérez-Ripoll, M., 2019. The early Upper Palaeolithic of Cova de les Cendres (Alicante, Spain). *Quaternary International*, 515, 92–124. <https://doi.org/10.1016/j.quaint.2017.11.051>.

Villaverde, V., Sanchis, A., Badal, E., Bel, M.Á., Bergadà, M.M., Eixea, A., Guillem, P.M., Martínez-Alfaro, Á., Martínez-Valle, R., Martínez-Varea, C.M., Real, C., Steier, P., and Wild, E.M., 2021. Cova de les Malladetes (Valencia, Spain): New Insights About the Early Upper Palaeolithic in the Mediterranean Basin of the Iberian Peninsula. *Journal of Paleolithic Archaeology*, 4(1), 5. <https://doi.org/10.1007/s41982-021-00081-w>.

Wood, R., Bernaldo de Quirós, F., Maíllo-Fernández, J.-M., Tejero, J.-M., Neira, A., and Higham, T., 2018. El Castillo (Cantabria, northern Iberia) and the Transitional Aurignacian: Using radiocarbon dating to assess site taphonomy. *Quaternary International*, 474, 56–70. <https://doi.org/10.1016/j.quaint.2016.03.005>.

Wood, R.E., Arrizabalaga, A., Camps, M., Fallon, S., Iriarte-Chiapusso, M.-J., Jones, R., Maroto, J., de la Rasilla, M., Santamaría, D., Soler, J., Soler, N., Villaluenga, A., and Higham, T.F.G., 2014. The chronology of the earliest Upper Palaeolithic in northern Iberia: New insights from L’Arbreda, Labeko Koba and La Viña. *Journal of Human Evolution*, 69, 91–109. <https://doi.org/10.1016/j.jhevol.2013.12.017>.

Zilhão, J., 1997. *O Paleolítico Superior da Estremadura portuguesa*. Lisboa: Colibri.

Zilhão, J., Anesin, D., Aubry, T., Badal, E., Cabanes, D., Kehl, M., Klasen, N., Lucena, A., Martín-Lerma, I., Martínez, S., Matias, H., Susini, D., Steier, P., Wild, E.M., Angelucci, D.E., Villaverde, V., and Zapata, J., 2017. Precise dating of the Middle-to-Upper Paleolithic transition in Murcia (Spain) supports late Neandertal persistence in Iberia. *Heliyon*, 3(11), e00435. <https://doi.org/10.1016/j.heliyon.2017.e00435>.

Zilhão, J., Davis, S.J.M., Duarte, C., Soares, A.M.M., Steier, P., and Wild, E., 2010. Pego do Diabo (Loures, Portugal): Dating the Emergence of Anatomical Modernity in Westernmost Eurasia. *PLOS ONE*, 5(1), e8880. <https://doi.org/10.1371/journal.pone.0008880>.
