# Supplementary material for: Unpacking lithic assemblage variability in the Early Upper Palaeolithic: A multivariate approach to the structure of the Iberian Aurignacian
Source: PLoS One. 2026 Mar 27;21(3):e0345202. doi: 10.1371/journal.pone.0345202 (PMC13028375; doi:10.1371/journal.pone.0345202)
Supplement: S1 Fig — The right column shows the final selection of attributes following the amalgamation of certain categories. (PDF) [file pone.0345202.s003.pdf]

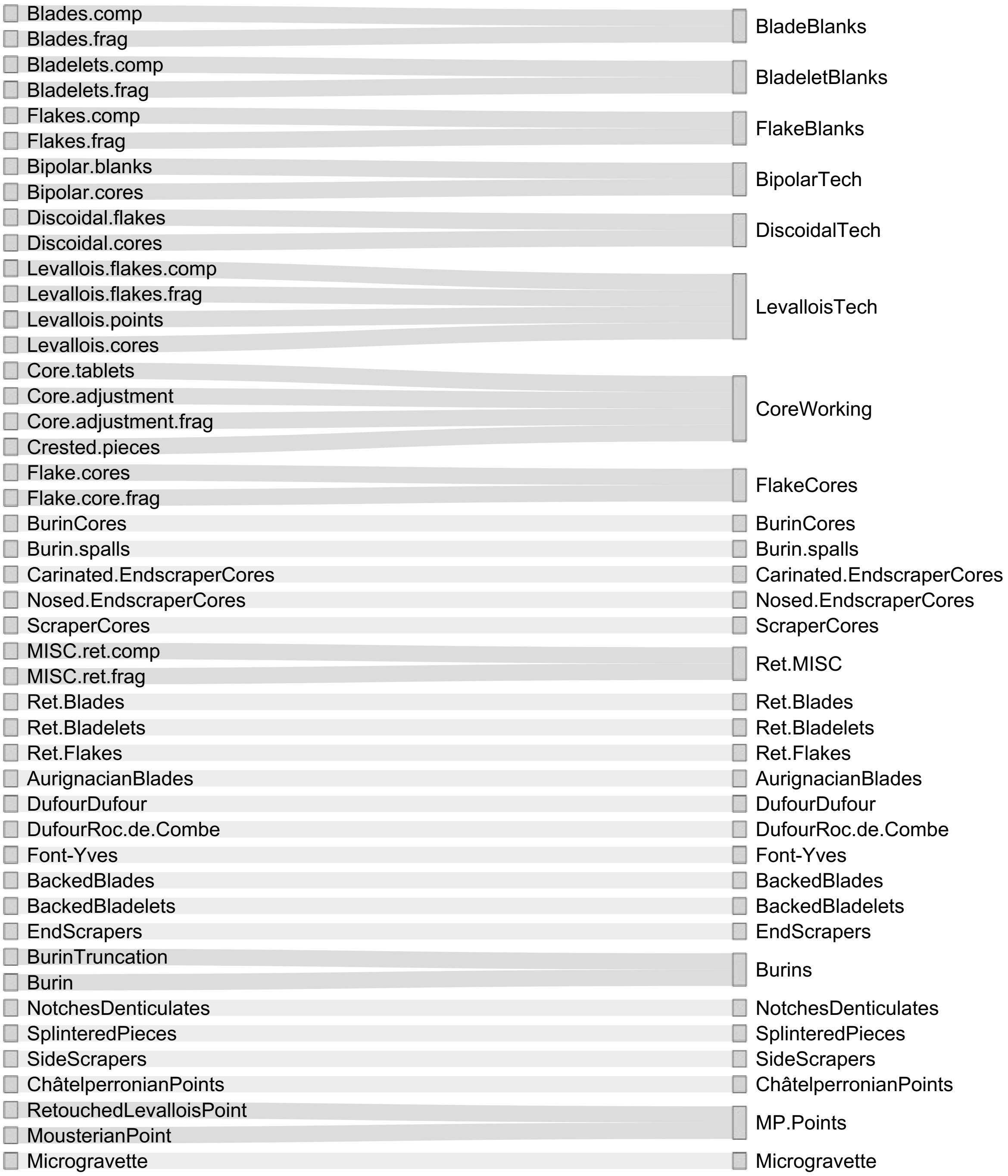

S1 Fig. Sankey diagram of techno-typological attributes analysed in this study. The right column shows the final selection of attributes following the amalgamation of certain categories
