# Supplementary material for: Unpacking lithic assemblage variability in the Early Upper Palaeolithic: A multivariate approach to the structure of the Iberian Aurignacian
Source: PLoS One. 2026 Mar 27;21(3):e0345202. doi: 10.1371/journal.pone.0345202 (PMC13028375; doi:10.1371/journal.pone.0345202)
Supplement: S2 Fig — Values in the heatmap range from 0 (identical presence of techno-typological attributes) to 1 (complete absence of shared techno-typological attributes). (PDF) [file pone.0345202.s004.pdf]

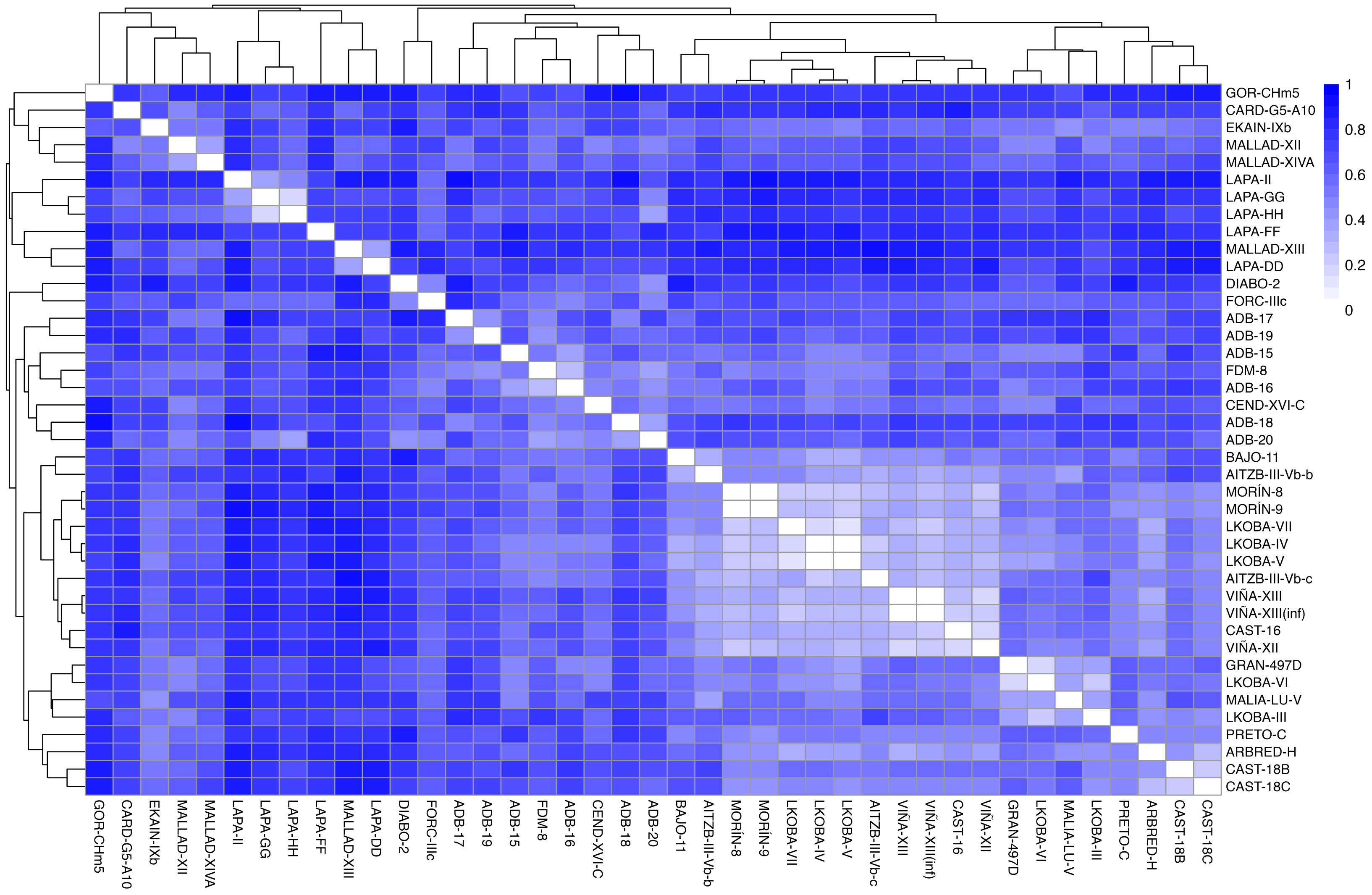

S2 Fig. Heatmap of Jaccard distance values between lithic assemblages. Values in the heatmap range from 0 (identical presence of techno-typological attributes) to 1 (complete absence of shared techno-typological attributes)
