# Supplementary material for: Unpacking lithic assemblage variability in the Early Upper Palaeolithic: A multivariate approach to the structure of the Iberian Aurignacian
Source: PLoS One. 2026 Mar 27;21(3):e0345202. doi: 10.1371/journal.pone.0345202 (PMC13028375; doi:10.1371/journal.pone.0345202)
Supplement: S1 File — (ZIP) [file pone.0345202.s005.zip › Data/READ_ME.rtf]

The two csv files in this folder comprise the raw lithic and chronological data needed for the analyses and plots carried out in R. The ‘Technocomplex’ column in each file provides the attribution given by corresponding publications, which in the case of Gorham’s Cave Chm.5 follows an adapted rewording of its original classification (see Table 1 of the paper for the sources). An exception to this rule concerns the category ‘possibly Aurignacian’ which we have applied to the below assemblages for the following reasons (see also ‘Materials and methods’ section of the paper for additional details):	▪El Castillo levels 18B and 18C – both levels contain diagnostic Aurignacian artefacts; avoidance of the loaded ‘Transitional Aurignacian’ label (Cabrera-Valdés et al., 1996)	▪Aitzbitarte level Vb-base – has Upper Palaeolithic techno-typological features; directly underlies a diagnostic Aurignacian horizon	▪Labeko Koba level III – no cultural attribution given; lacks diagnostic artefacts but directly overlies an Aurignacian horizon	▪Lapa do Picareiro level DD – no cultural attribution given; lacks diagnostic artefacts but dated to the Aurignacian timeframe	▪Lapa do Picareiro level FF – no cultural attribution given; lacks diagnostic artefacts but directly overlies an Aurignacian horizonEach csv file is also the basis for S1 and S2 Tables which are provided separately as processed data (xlsx files). The code for the creation and export of these tables can also be found in the R script (Script.R).
