# Supplementary material for: Unpacking lithic assemblage variability in the Early Upper Palaeolithic: A multivariate approach to the structure of the Iberian Aurignacian
Source: PLoS One. 2026 Mar 27;21(3):e0345202. doi: 10.1371/journal.pone.0345202 (PMC13028375; doi:10.1371/journal.pone.0345202)
Supplement: S2 File — (DOCX) [file pone.0345202.s006.docx]

**Supplementary analyses and figures**

The below figures and tables present the results of Correspondence Analysis (Part 1) and partial Mantel tests (Part 2) following a more stringent selection of techno-typological attributes and radiocarbon dates (Section 6 of R script). The attributes excluded from these supplementary analyses are *LevalloisTech*, *ChâtelperronianPoints,* *MP.Points* and *Microgravettes* (Fig 2 of manuscript and S1 Fig in supporting information), as these are characteristically non-Aurignacian traits that may generate unwanted noise. *DiscoidalTech* is nonetheless included due its more frequent and temporally expansive occurrence (see also Discussion section of manuscript for further details).

Concerning partial Mantel tests, the more stringent selection of radiocarbon dates was based on AMS measurements and more rigorous pretreatment methods (i.e., ABA, ABOx-SC and ultrafiltration protocols). As the only available date for La Viña level XIII(inf.) comes from a conventional C14 measurement (rather than AMS), it is retained in the analysis to preserve the sample of dated assemblages. It should anyhow be noted that its calibrated age aligns with the chronology of the Proto-Aurignacian.

In all the given cases, the results of Correspondence Analysis and partial Mantel tests support those presented in the manuscript.

**Part 1 – Correspondence Analysis**

**
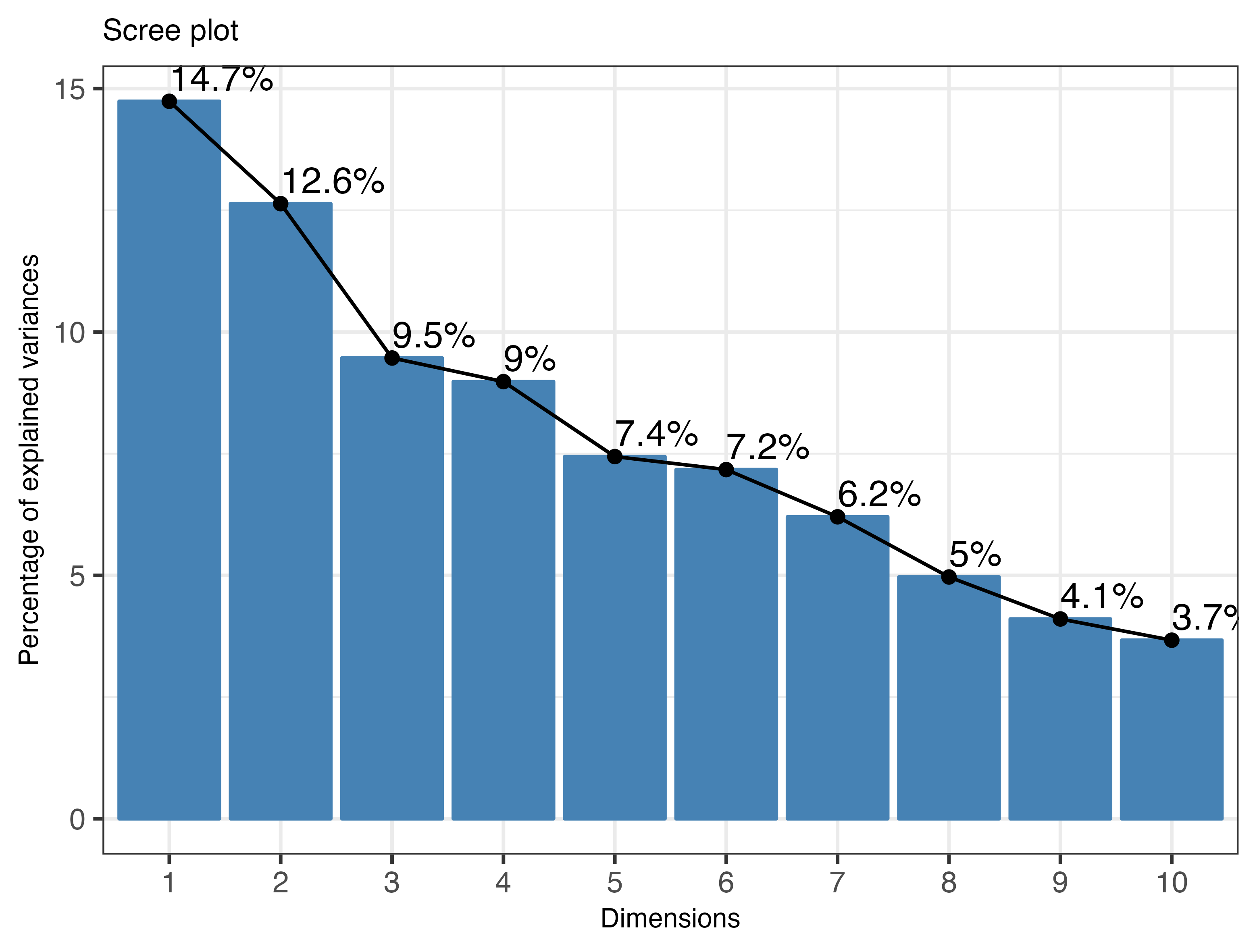
**

**Fig 1.1. Scree plot showing the contribution of each dimension to the explained variance after the removal of non-Aurignacian attributes**

**
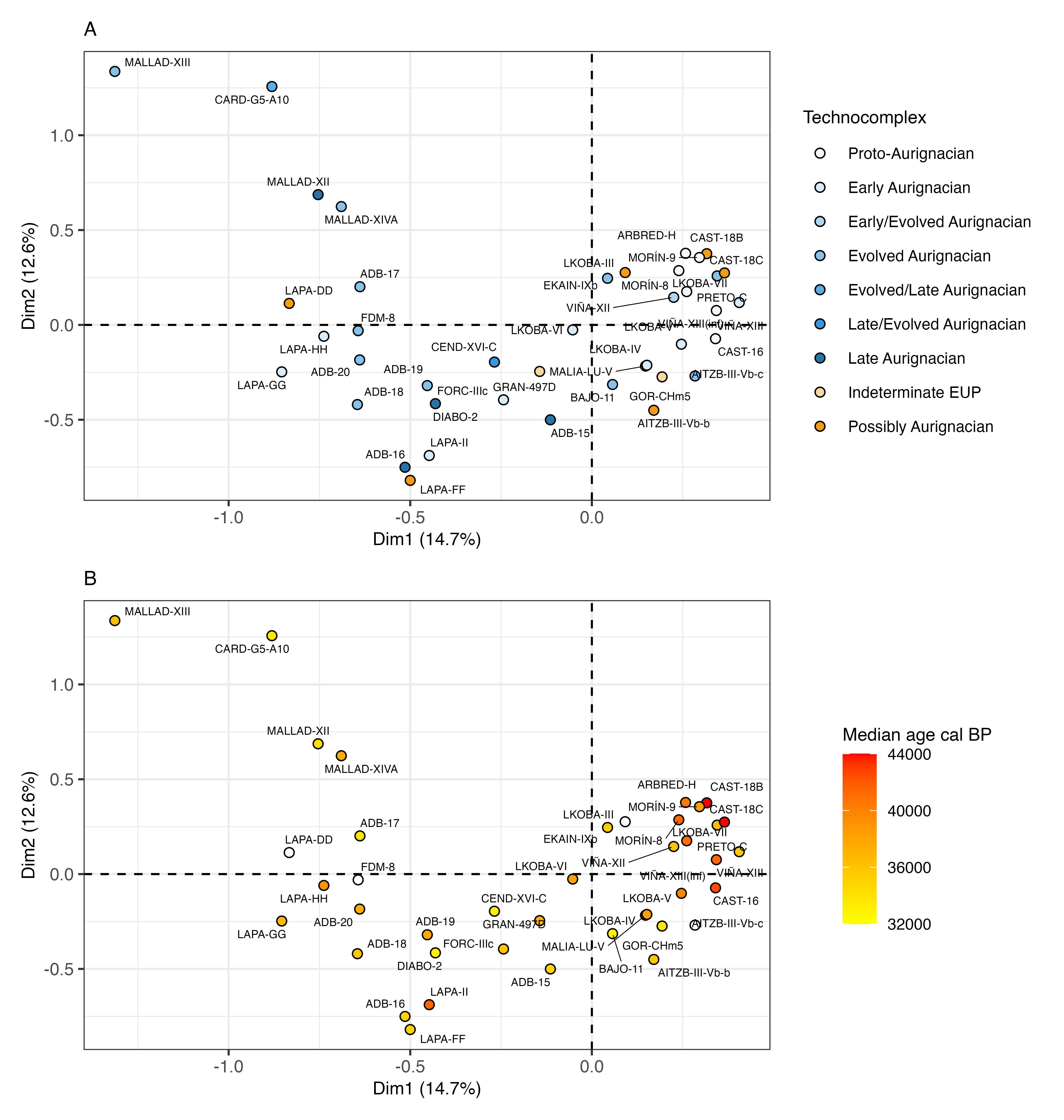
**

**Figure 1.2. Correspondence Analysis row plots of the first two dimensions after the removal of non-Aurignacian attributes.** A) Row plot of sites coloured by technocomplex and B) row plot of sites coloured by median calibrated ages BP (the four undated assemblages are coloured white)

**
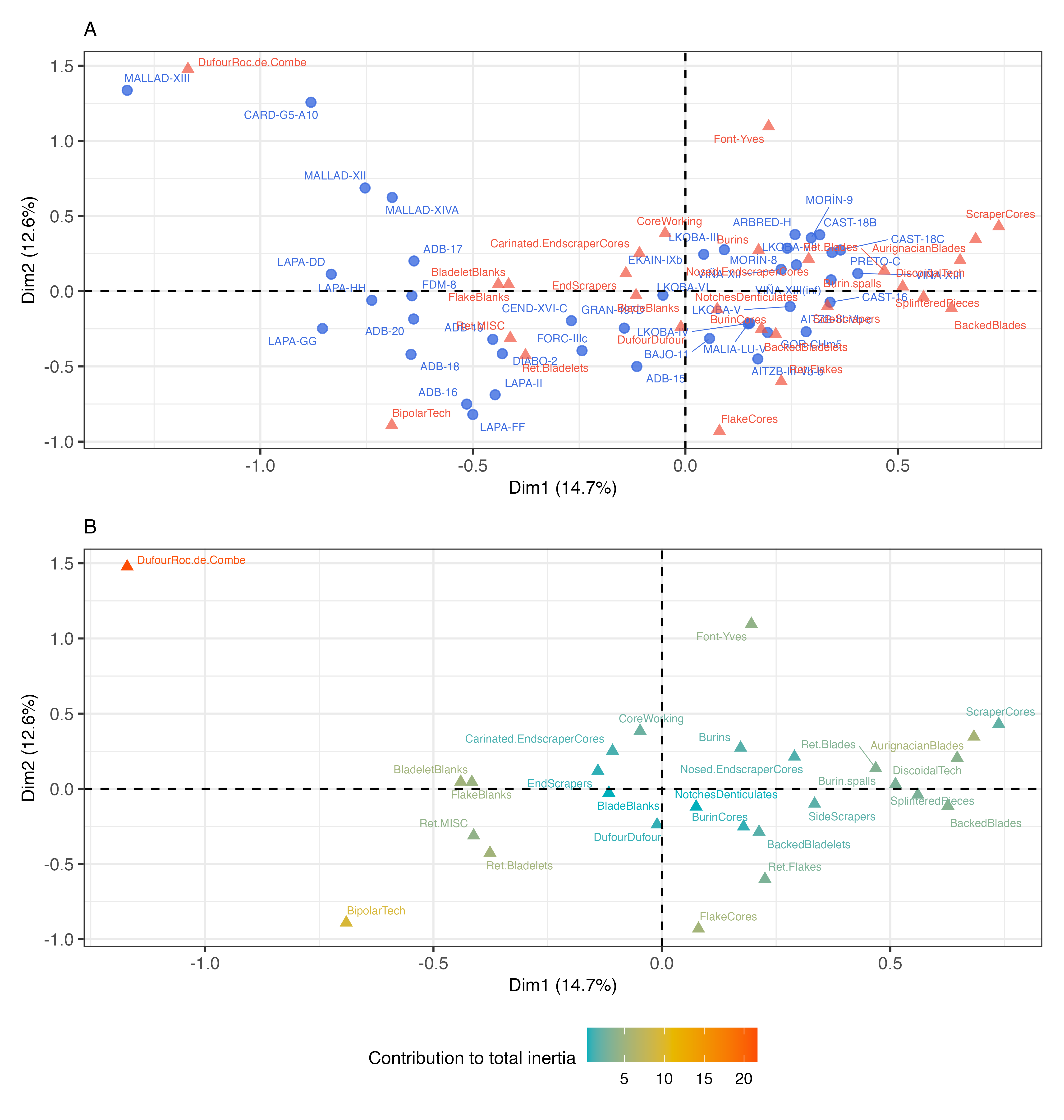
**

**Figure 1.3.** **Correspondence Analysis biplot and column plots after the removal of non-Aurignacian attributes.** A) Combined biplot of rows (sites) and columns (techno-typological attributes) and B) column plot showing the contribution of variables (techno-typological attributes) to the variance explained by the two dimensions. The colour ramp indicates the contribution as a percentage.

**
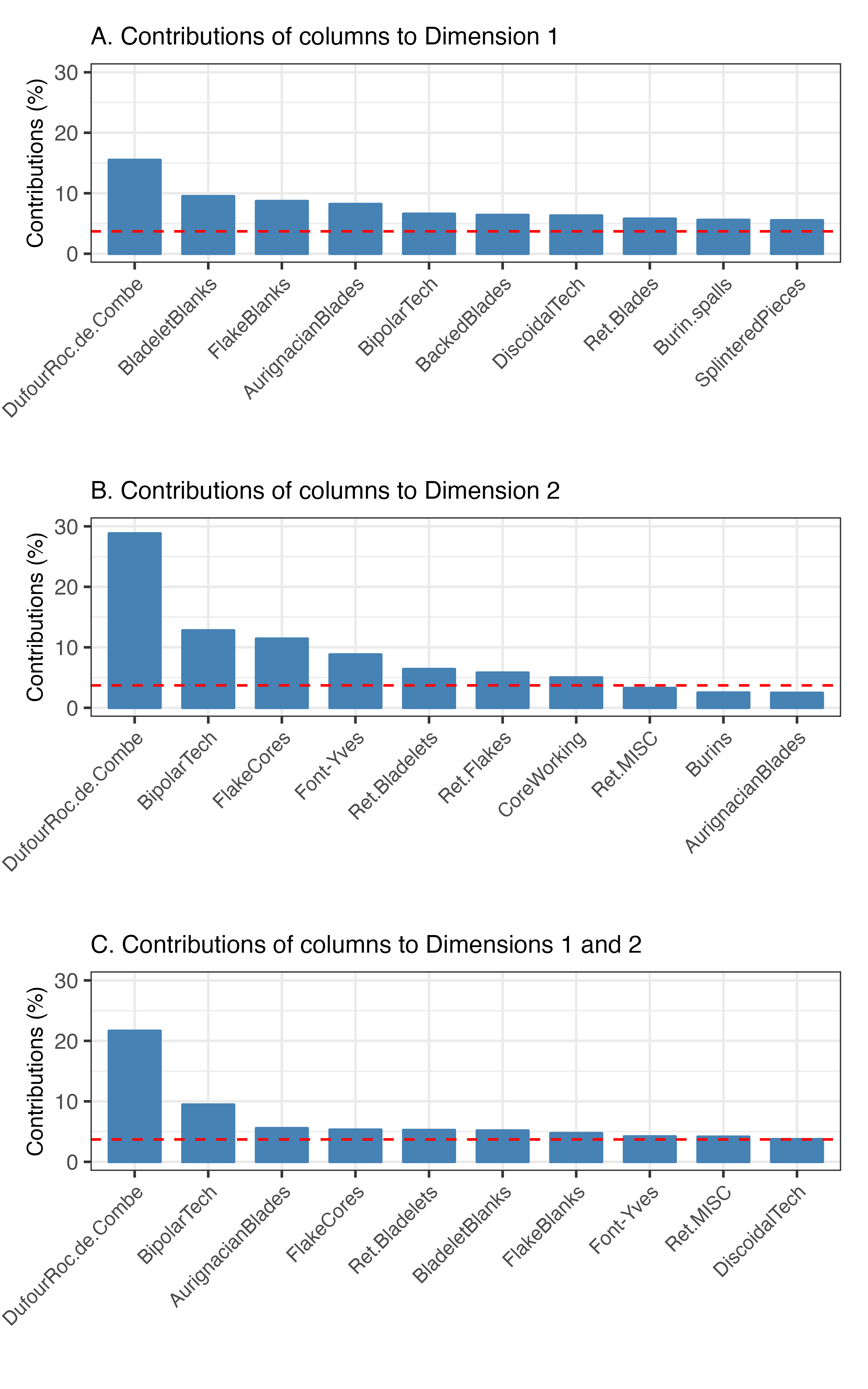
**

**Figure 1.4. Bar graph showing the contributions of column variables (techno-typological attributes) to the first two dimensions after the removal of non-Aurignacian attributes.** A) Contributions to the first dimension, B) contributions to the second dimension and C) Combined contributions to the first and second dimensions.

**Summary**

Even after the removal of non-Aurignacian attributes, the distribution of assemblages in a row plot (Figure 1.2) reflects the general spatial pattern observed in the original analysis. While assemblages can be seen to shift slightly across each axis, the general distribution is nonetheless maintained. Likewise, the association between assemblages and attributes supports the observation that certain attributes are less discriminating variables, as per the position near the centre of the plot (e.g., *BladeBlanks, NotchesDenticulates, Endscrapers, DufourDufour* and to a lesser extent, *Carinated.EndscraperCores, Burins* and *CoreWorking* elements) (Figure 1.3). However, compared to the original analysis, some of these attributes edge further away from the centre (e.g., *CoreWorking*) whilst others draw slightly nearer (e.g., *BurinCores*). More discrete tool types again show stronger associations to northern assemblages that plot to the right of the central axis even though the number of typological attributes is reduced.

*DufourRoc.de.Combe* and *BipolarTech* remain the most significant overall contributors to the first two dimensions and are again found at the extremities of the plot (Fig 1.2 and Fig 1.3). However, this time their scores are slightly higher following the reduction of analysed attributes. The rest of the attributes have little to no contributions above the value expected if all contributions were to be equal (red-dotted line in Fig 1.3). *FlakeCores* appear to have a greater contribution to the second dimension but its overall contribution to the first two dimensions is near equal to that seen in the original analysis. Its increased ranking could relate to the removal of *ChâtelperronianPoints,* as this column variable was previously the third greatest contributor to the variance explained by the second dimension. In connection, the contribution of *BipolarTech* to the second dimension is seen to increase slightly when compared to the original analysis, making it the second largest contributor and therefore a greater component of the variance expressed by this dimension.

**Part 2 – Partial Mantel tests**

**Table 1.1. Results of partial Mantel tests between the three distance matrices based on radiocarbon samples subject to more rigorous pretreatment methods**

| **Distances matrices** | **Statistic** | ***p*-value** | **MSR *p-*value** | |
| --- | --- | --- | --- | --- |
| Jaccard distance and spatial distance, controlling temporal distance | 0.3508 | **0.0001** | **0.0184^a^** | **0.0195^b^** |
| Jaccard distance and temporal distance, controlling spatial distance | -0.03069 | 0.6191 | – | – |

^a^ Following spatial weights based on K=5

^b^ Following spatial weights based on K=10

**Table 2.1. Results of partial Mantel tests between the three distance matrices after the removal of non-Aurignacian attributes**

| **Distances matrices** | **Statistic** | ***p*-value** | **MSR *p-*value** | |
| --- | --- | --- | --- | --- |
| Jaccard distance and spatial distance, controlling temporal distance | 0.3465 | **0.0001** | **0.0131^a^** | **0.0164^b^** |
| Jaccard distance and temporal distance, controlling spatial distance | -0.03528 | 0.6512 | – | – |

^a^ Following spatial weights based on K=5

^b^ Following spatial weights based on K=10

**Table 2.2. Results of partial Mantel tests between the three distance matrices after the removal of non-Aurignacian attributes using only samples subject to more rigorous pretreatment methods**

| **Distances matrices** | **Statistic** | ***p*-value** | **MSR *p-*value** | |
| --- | --- | --- | --- | --- |
| Jaccard distance and spatial distance, controlling temporal distance | 0.3473 | **0.0001** | **0.026^a^** | **0.0336^b^** |
| Jaccard distance and temporal distance, controlling spatial distance | -0.04852 | 0.7082 | – | – |

^a^ Following spatial weights based on K=5

^b^ Following spatial weights based on K=10

**Summary**

The strength and significance of correlation between Jaccard distance and spatial distance is the same across all three analyses and additionally replicates the originally obtained result. Likewise, in line with the original result, there is a lack of correlation between Jaccard distance and temporal distance.
