# Supplementary material for: Unpacking lithic assemblage variability in the Early Upper Palaeolithic: A multivariate approach to the structure of the Iberian Aurignacian
Source: PLoS One. 2026 Mar 27;21(3):e0345202. doi: 10.1371/journal.pone.0345202 (PMC13028375; doi:10.1371/journal.pone.0345202)
Supplement: Attachment — Submitted filename: Response to Reviewers.pdf [file pone.0345202.s008.pdf]

12th February 2026

Dear Academic Editor,

We greatly appreciate the consideration of our Manuscript in PLOS ONE and the time invested by the three anonymous reviewers. We take note of the points raised in the opening paragraph of the decision letter that refer to the study's 'sampling criteria', 'data structure and composition', 'data analysis (CA)' and 'use of terminology'. While we have addressed individual reviewer comments in a later section of this letter, we nonetheless inform you that these individual elements have been tackled in the following way:

Sampling criteria:
We have addressed comments about the presence-absence format of lithic data and provided additional text in the Manuscript that highlights this potential limitation. Moreover, we have given greater emphasis to the fact different patterns could be observed with analyses of continuous variables (e.g., proportion of core types or bladelets) rather than the categorical variables we use in our study. We have also run additional analyses with different sample selection criteria for both techno-typological attributes and radiocarbon dates. These have been referenced in the Manuscript and presented separately in a new S2 File.

In all cases these additional analyses with different criteria replicate the original results of the Manuscript.

Data structure and composition:
In line with the above, we have addressed concerns that the data may be too typologically driven and provided the results of further analyses with less typological attributes in the S2 File. Specific comments on the significance/insignificance of certain techno-typological attributes have also been addressed via individual responses.

Data analysis (CA):
Overlapping with the above, the results of a CA without potentially intrusive or characteristically non-Aurignacian attributes (i.e., *LevalloisTech, ChâtelperronianPoints, MP.Points and Micogravettes*) are mentioned in the Manuscript to support the original conclusions. The plots are additionally provided in the S2 File along with some text summarising the analyses.

Use of terminology:
Terminological issues raised by Reviewers 1 and 3 have been addressed, the details of which can be found in our responses. We have also refined the use of other terms not specifically highlighted.

Other:
We noticed a labelling error in Fig 1 and have now submitted a corrected version. Other typos or mistakes recognised during our rereading of the Manuscript have also been amended.

With regards to the Journal Requirements listed in the letter, we hereby confirm that each requirement has been adhered to in accordance with the following:

'1.Please ensure that your Manuscript meets PLOS ONE's style requirements, including those for file naming.'

We confirm that the Manuscript meets all of PLOS ONE's style requirements. As per Editorial Office's request, we have amended the referencing style.

'2. In your Manuscript, please provide additional information regarding the specimens used in your study. Ensure that you have reported human remain specimen numbers and complete repository information, including museum name and geographic location.'

No specimens were used in our study.

'3. When completing the data availability statement of the submission form, you indicated that you will make your data available on acceptance. We strongly recommend all authors decide on a data sharing plan before acceptance, as the process can be lengthy and hold up publication timelines. Please note that, though access restrictions are acceptable now, your entire data will need to be made freely accessible if your Manuscript is accepted for publication. This policy applies to all data except where public deposition would breach compliance with the protocol approved by your research ethics board. If you are unable to adhere to our open data policy, please kindly revise your statement to explain your reasoning and we will seek the editor's input on an exemption. Please be assured that, once you have provided your new statement, the assessment of your exemption will not hold up the peer review process.'

We have created a Zenodo entry and attached its doi to the data availability statement. This doi link will be activated if/once the manuscript is accepted for publication, as we do not want our data to be used before the associated study is actually published. This follows the offer stated above, which permits restricted access until the Manuscript is accepted for publication.

'4. We note that Figure 1 in your submission contain map images which may be copyrighted. All PLOS content is published under the Creative Commons Attribution License (CC BY 4.0), which means that the Manuscript, images, and Supporting Information files will be freely available online, and any third party is permitted to access, download, copy, distribute, and use these materials in any way, even commercially, with proper attribution. For these reasons, we cannot publish previously copyrighted maps or satellite images created using proprietary data, such as Google software (Google Maps, Street View, and Earth). For more information, see our copyright guidelines: http://journals.plos.org/ plosone/s/licenses-and-copyright.'

We have amended Figure 1 following guidance from the Editorial Office. The Digital Elevation Model is now taken from the free to use USGS EROS Centre (https://www.usgs.gov/centers/eros/data). Use of the ocean bathymetry data (from the EMODnet) remains unchanged as it is licensed under Creative Commons CC-BY 4.0 (see https://emodnet.ec.europa.eu/en/terms-use-emodnet-online-services-data-and-data-products). The relevant figure caption has also been amended.

'5. If the reviewer comments include a recommendation to cite specific previously published works, please review and evaluate these publications to determine whether they are relevant and should be cited. There is no requirement to cite these works unless the editor has indicated otherwise.'

We confirm that suggestions to cite specific published works have been reviewed and evaluated with accuracy.

With this said, we would like to thank the three anonymous reviewers for their constructive and insightful comments which have helped improve the Manuscript. We are especially grateful for the positive evaluation of our work and identification of certain weaknesses. Below we address each

comment with a response in blue text and refer to line numbers from the revised Manuscript. Please note that the line numbers in Microsoft Word been observed to shift after saving the document so the numbers provided here may be marginally incorrect.

Reviewer #1: Review
The article "Unpacking lithic assemblage variability in the Early Upper Palaeolithic: A multivariate approach to the structure of the Iberian Aurignacian" authored by T. Canessa and P. de la Peña, presents a quantitative approach based on techno-typological data from Aurignacian sites in Iberia. The authors applied multivariate statistics to lithic datasets to characterise the spatial and temporal structure of the Aurignacian in Iberia. This work aims at identifying if there are inter-assemblage variability, both diachronic and synchronically among Aurignacian sites in Iberia, by applying a previously non-explored quantitative approach. The article introduces in detail the topic of study, first with the Aurignacian itself and then with the Early Upper Palaeolithic in Iberia, exhibiting extensive knowledge of the topic. The figures are pertinent, helping to understand the text and clarifying the main aspects of the Manuscript. The text is well written, without typos, and with a good exposition of the data. The authors provide an Rscript and all the rawdata for the replicability of the study, aligning with Open Science practices which are well valuated in research community. All this makes it an important contribution to the field of archaeological research. However, there are certain issues that concerns me regarding the methods, the quality of the data and the interpretation of the results.
I therefore consider these changes, especially those concerning the data composition and the data analysis (CA), which are fundamental to support the hypothesis and final interpretations of the author, must be addressed.
I will explain my main concerns here followed by some minor comments at the end of the review.

We appreciate the recognition of our work and its contribution to open science. We have addressed the issues raised regarding its methods, the quality of the data and interpretation below under individual comments.

Introduction
I would rework the introduction ensuring some clarity. It starts with the IUP, although there are not much information about it, then it could start directly from the Aurignacian (AU since now in advance) as it is the topic of this work. The explanation of the AU (Line 52) should have some of the references mentioned below (Line 58). But more importantly, the authors should note that the fours subdivisions of the AU are already explained by G.Laplace (1966) and then it should be the main reference used. Why it is only used the framework of Aquitaine and not the one from the Italian Peninsula, or the core sequence of Central Europe (Swabian Jura, Moravia..)
Also, as a suggestion, perhaps the authors could be more selective with literature to avoid overciting (81-83; 97-101).

Our brief mention of IUP assemblages serves to provide context to the Aurignacian of the subsequent EUP. This is because, in contrast to the circumscript nature of IUP assemblages (not all of which are currently and exclusively associated with *Homo sapiens*), the Aurignacian is a technocomplex with extensive geographic and chronological distribution. We therefore aimed to emphasise that this is one of the qualities which renders it a topic of enduring interest in Palaeolithic archaeology. To this end, we have reworked the opening paragraph to make this point clearer.

With regards to work of G. Laplace (1966), he divided the Aurignacian into four main phases (*Protoaurignacien, Aurignacien Ancien, Aurignacien Évolué* and *Aurignacien Évolué Final*) and suggested further subdivisions for most of these based on their typological compositions e.g.

Aurignacien Évolué à fiable indice de burins and *Aurignacien Évolué à fort indice de burins.* While we acknowledge his work as an important contribution, we are inclined to argue that it is not the main reference for the Iberian Aurignacian. The four subdivisions we list in line 68 are the naming conventions mostly commonly used in the Iberian literature. These are collectively based on the the many works we cite in the paragraph and recently refined with archaeological and chronometric evidence. To make this clearer, we have added the word 'consolidation'.

The Aquitaine framework is referred to as the leading framework because is the one that has traditionally informed upon the identification and characterisation of the Iberian Aurignacian (e.g. Zilhão 1997, Villaverde et al. 1998), not least because of the proximity to SW France and the influence of French scholarship on Palaeolithic research in Iberia. Therefore, for our study and its questions, we believe that the frameworks derived from Italy or Central Europe do not supersede the Aquitaine one.

We accept the suggestion of being more selective with citations and have done so. We have also added Gennai et al. 2025 following Reviewer 3's suggestion.

Line 111: "how do we reconcile this variability with the notion that the Aurignacian is a shared tradition? " This question is ambiguous to me, as variability has always existed in lithic assemblages and it is recognised within cultures. Another question is, what is and atypical assemblage? Atypical with respect to what? (Line 112). In my opinion, this term is rooted in a deep typological perspective, in which lithic assemblages were explained based on diagnostic artefacts. I recommend to reconsider using this terminology. If the authors aim at identifying variability among assemblages, then, atypical should not be used.

We agree that variability has commonly been observed within lithic industries, even when some influential scholars have emphasised a more homogenous, pan-European picture of industries. What has been less explored, however, is whether the extent of this variability lends support to the idea that the Iberian Aurignacian is a strongly represented and shared tradition (a point we later address in the Discussion). We have now modified the wording to make this clearer.

We also welcome the question 'what is an atypical assemblage?' as a valid one. In our question posed in the Introduction, we are referring to assemblages which are atypical in the cultural taxonomic sense. Of course, from a behavioural perspective 'atypical' is misleading. We have hence replaced the term 'atypical' throughout the paper.

Methods

The authors explained they used 41 datasets from the Early Upper Palaeolithic but they should mention which were the criteria used for the selection of data. Do they collect all the available datasets in the literature? Were some sites excluded? If so, what was the criteria used to not include those sites? The authors should detail these aspects.

We appreciate if the criteria of assemblage selection was not fully clear based on the information provided in lines 247–284. For the avoidance of doubt, we aimed to include as many assemblages as possible that are archaeologically and/or chronologically relevant to the Aurignacian in Iberia; in other words, assemblages claimed to be Aurignacian based on their lithic artefact component or assemblages dated to the Aurignacian timeframe but which have been classified differently (e.g. 'indeterminate Early Upper Palaeolithic') or not at all (e.g. Lapa do Picareiro). In connection, as our analysis is concerned with both the technological *and* typological composition of these assemblages, only assemblages with sufficient published data were selected (it is for this reason that the Abstract states "all sufficiently published and chronologically relevant assemblages"). This means that, for example, assemblages with only retouched tool

counts published could be not included in the analysis for lack of technological information (for publications from the 20th century, this is unfortunately a common occurrence).

In parallel to this, another major concern is the quality of the data collected. Specially in the election of the sites or the comparable attributes.
Concerning the sites, for example, level 18b and 18c of El Castillo has been already attributed to Neanderthals (Garralda et al. 2022) because of the presence of three Neanderthal deciduous teeth, excluding the possibility of anatomically modern humans as the makers of this assemblage and so, its Aurignacian adscription. Level CHm.5 of Gorham's Cave reported 17 artefacts in a disputable context. Same occurs in Aitzbitarte Vb where Aurignacian-Mousterian mixing was identified in the upper layer. Level XIIIinf of La Viña has been affected by post-depositional processes resulting in mix with Middle Palaeolithic units (Santamaria, 2012). Pego do Diabo is another controversial site. Excavated by spits, its level 2 lies between the Gravettian and Aurignacian attribution, but no clear evidence of the last one. One might wonder to what extent these examples can distort the subsequent analysis. In mi opinion, level 18b and 18c from El Castillo must be removed.

Respectfully, we do not agree with the idea that all archaeological materials of levels 18B and 18C in El Castillo can be exclusively attributed to Neanderthals based on metric and qualitative attributes (rather than genetic or proteomic evidence, or the morphology of the internal structures of the teeth) of three worn deciduous tooth crowns. Indeed, to exclude the possibility of an anatomically modern human component would assert that the Neanderthals were exclusively responsible for the characteristic Aurignacian (e.g. bone tools, carinated- and nosed-endscraper cores, Aurignacian blades and Dufour bladelets [Fig. 2]) and general Upper Palaeolithic elements like blades, bladelets and end-scrapers. As stated in the Manuscript, further analyses of the deposits and its materials need to be carried out in order to ascertain the authorship of the assemblage. Therefore, before this is achieved, it remains a valid hypothesis that that these levels are equally the product (whether partially or entirely) of anatomically modern humans. It is for this reason that we also classify levels 18B and 18C as 'possibly Aurignacian'.

Similarly, we do not find evidence to consider level CHm.5 of Gorham's Cave as a disputable context. If this view is based on the paucity of lithics, it should be known that the size of the assemblage is related to the few deposits that remained after John Waecther's extensive excavations in 1948-54. From the available lithic and geoarchaeological studies published in the 2012 monograph of the relevant excavation (Barton et al. 2012), there is no indication that the level CHm.5 is heavily reworked. Moreover, the artefacts derive from a level with combustion features and there is a good association between the artefacts and dated samples (charcoal).

We do of course agree that El Castillo 18B and 18C, Aitzbitarte III level Vb, La Viña level XIII-inf and Pego do Diabo have their problems, like all Palaeolithic sites do. However, rather than remove them from the analysis, we now present the results of additional analyses in the S2 File that excludes the attributes deemed as non-Aurignacian: ChâtelperronianPoints, MP.Points, LevalloisTech and Microgravette. In all cases the CA and Mantel tests replicate the original results.

The authors could provide alternative analysis with a better-chosen sample of sites, or at least after removing the ones highly controversial for its chronocultural attribution (e.g. Castillo, Morin, Gorham's, Pego do Diabo, etc.). This new analysis could be cross compared to see if the final results excluding controversial sites match with the ones they have already obtained.
Also, the authors should clarify the distinction they do about north and south. And why they preferred to use this distinction among others base on the biogeographical space (Vidal-Cordasco et al. 2022)

We thank the reviewer for suggesting an alternative analysis with a more cautious sample of sites. While insightful, we believe this would change the primary objective of the study, which is to test the full spectrum of assemblages that fall within the timeframe of the Aurignacian. Therefore, as above, instead of removing sites and limiting the studied sample, we have chosen to control for the effect of unwanted noise by presenting additional analyses that removes potentially intrusive or characteristically non-Aurignacian variables (provided in S2 File).

We agree there is a lack of detail on the north-south distinction. In our the commentary of our results, we are referring to a simple geographical north beginning 40°N, and which divides the peninsula into almost equal halves, as now mentioned in line 575.

I would like to know if the authors have considered possible discrepancies with some dates of the assemblages that has low agreements in order to calculate the median. And to what extent the temporal variable can be the result of different lab methods or treatments.

Thank you. Our original analysis of median ages for each assemblages was carried out following the exclusion of unreliable and problematic C14 dates (see columns 'Accepted' and 'Reason' in 'Raw_dates.scv' and R script, Section 2) . However, in the S2 File we now present the results of additional analyses whereby only ABA, ABOx-SC and the ultrafiltration protocol (for bone collagen) are used. These results are presented in a supplementary document because they effectively replicate the original ones. The code for these analyses is also included in the updated R script.

Concerning the attributes, the application of multivariate statistics is good for make the data homogeneous. However, I am concerned about which data is included in the analysis and if these choices are contributing to biased results. First, the attributes are more typological than technological, and it can be seen in the Discussion, where most of the insights are based on the presence of retouched tools from a typological perspective, which is applying the same perspective the authors are trying to overcome/solve.

This is a valid point. We tried to maximise the amount of technological attributes but the variability of published data makes this a difficult task. Most publications are indeed typological, meaning that technological data is either absent or insufficient for the type of analyses we have carried out. Nevertheless, in order to compensate for a potential imbalance – and in line with some above suggestions – the additional analyses we provide in the S2 File include less typological variables.

Regarding the Discussion, we do not feel that we are necessarily applying the same typological perspective that underpins many other publications. Rather, when discussing the significance of typological variability we are highlighting (based on our collected data (Fig 2) and results) that the time-sensitive nature of typological index fossils may not be supported. Moreover, the discussion of technology is limited because of the presence-absence format of the data: it is therefore beyond the scope of the paper (nor does the data necessarily allow) for us to discuss differences in reduction sequences, core management strategies and technological choices represented by the assemblages. Having said that, we have now expanded on the significance of bipolar and discoidal schemes of flaking in the Discussion following a suggestion made by Reviewer 3.

Second, I wonder how comparable assemblages over 5000 artefacts, 1000 artefacts or less than 100 artefacts can be? Do they have the same relevance? From my perspective, 1 Dufour bladelet (which already means presence) over 5000 artefacts should not represent the same than 1 Dufour bladelet over 60 artefacts. And here it can be also discussed different occupation models and interpretations of the assemblages.

Yes, this is an important point. In the Manuscript we have emphasised that the presence-absence data sacrifices a fine-grained resolution for a larger sample of assemblages, among other things. We have now added some additional commentary on this implication in lines 933-937. We would also like to emphasise that we have worked with the data that exists – because of the nature of published data, a more nuanced or in-depth analysis cannot carried out on a such a large sample of EUP Iberian assemblages.

Furthermore, it is our goal, in the future, to perform multivariate statistical analyses on continuous variables such as ratios of core types etc. (see also comments from Reviewer 3 on this matter) even though this type of analysis would reduce the sample of assemblages. This submitted paper is an initial step at synthesising the Iberian Aurignacian and testing its spatial and temporal variability.

Third, fossil directors are always reported in techno-typological studies, but not all the variables the authors gathered. Even though, they tried to concentrate different name conventions within a single term appropriately. I have these concerns with bipolar technology (addressed by the authors but included in the analysis), flake cores (which kind of reduction?). Also, flake core, burin core and carinated cores are not gathering the variability of reduction strategies in the EUP or AU and it is a weakness in the analysis. And last, did the authors consider the data that were missing in the original datasets? Did they account them as "not present"? This might revealed different results.

Our goal is not to capture the variability of reduction strategies with a presence-absence dataset. Rather, we have set out to explore and test the spatio-temporal variability of techno-typological traits.

With regards to flake cores, not all publications specify the kind of reduction nor can all flake cores be always attributed to a single type of reduction. Therefore, we opted to include a generic category (FlakeCores) that subsumed the variability of reported flaking techniques (not including Levallois, discoidal or bipolar). In our view, it was better to have a generic category that captures non-Levallois/discoida/bipolar flake reduction than not, as these types of flake cores represent interesting technological variability in the Aurignacian (which is conventionally seen as blade/bladelet industry).

In lines 366-368 we explained that if a specific artefact type was not reported in a publication, we treated it as being absent. However, as we only selected publications that had well reported technological and typological data, we are not under the impression that this is a limiting factor in our study.

If the authors applied any strategy to mitigate inter-analyst biased or to increase lithic replicability, they should also report it in the method section (Pargeter et al. 2023).

Strategies to mitigate inter-analyst biases are not applicable to our study. This is because the lithic techno-typological data we analyse has been directly obtained from publications in accordance with the way it is published. This means that, on a consistent basis, the data has not been subject to any transformations before entering our database. For example, as explained in lines 423-427 an artefact published as as splintered piece has been recorded in the dataset as a splintered piece, despite the fact that it may indeed be a bipolar core.

Finally, the methods are considerably long and I suggest the authors condense the attributes presented in this section in the main text and set the details in a supplementary document. This will increase readability of the main points of the article and give place to explain what a Correspondence Analysis is (no need to explain it in the article as it is a worldwide term used in research)

We think the methods section, including the presentation of attributes, is important contextual information that we would like to retain in the main text. However, we have now provided a Sankey diagram as a supplementary element (S1 Fig) to show the the grouping of attributes in a visual way. Also, we have also shortened the paragraph on CA as per your suggestion, thank you.

Garralda, MD., Maíllo-Fernández, JM., Maureille, B. et al. >42 ka human teeth from El Castillo Cave (Cantabria, Spain) Mid-Upper Paleolithic transition. Archaeol Anthropol Sci 14, 126 (2022). https://doi.org/10.1007/s12520-022-01587-2
Pargeter J, Brooks A, Douze K, et al. Replicability in Lithic Analysis. American Antiquity. 2023;88(2):163-186. https://doi.org/10.1017/aaq.2023.4
Vidal-Cordasco, M., Ocio, D., Hickler, T. et al. Ecosystem productivity affected the spatiotemporal disappearance of Neanderthals in Iberia. Nat Ecol Evol 6, 1644–1657 (2022). https://doi.org/10.1038/s41559-022-01861-5

Results
The authors reported very interesting results suggesting that Aurignacian assemblages in Iberia are highly variable and those differences can be tracked spatially but not diachronically. One special mention is to the identification of some discrepancies in the chronological order of chronocultural markers as Dufour subtype Dufour bladelets and Dufour subtype Roc de Combe bladelets. They expand these ideas in the discussion, also mentioning that probably, the reference we used for structuring the Aurignacian are not that consistent than previously thought. However, although these results are interesting and deserve to be further explored, I am not sure how reliable the author interpretations are looking at the CA analysis. The sum of the two dimensions account to 24%. This is a limited representation of the total contributions of the variables. Although the Eigenvalues are quite low in the 3, 4, 5, 6 dimensions, they could also represent alternative CA to see if the trends identified can be also supported by a more complete analysis of the data.

The sum of the first two dimensions accounts to 24% due the number of dimensions analysed and only increases to 27% when more attributes are removed (now line 557 and new S2 File). We wish to emphasise that falsifiable evidence for a spatial pattern and lack of a temporal pattern essentially comes from partial Mantel tests. CA is exploratory and is therefore only suggestive of a these patterns based on the distribution of assemblages in a row plot of dimensions 1 and 2. In our Conclusion we state that the results from the Mantel tests corroborate the suggested patterns shown by CA.

The authors should provide CA of the other dimensions (e.g. 3 and 4; 5 and 6; 3 and 5, 1 and 3) to make their interpretations stronger. This is relevant as trends observed the first and second dimensions can differ from other identified in the third, the fourth or the fifth.

We agree that trends observed in subsequent dimension can be different, however the eigenvalues of 3-6 are relatively low and therefore much less informative of underlying associations. It is for this reason why the focus of our commentary is on the first and second dimensions, as these are the most significant ones in a CA. Moreover, as explained above, the use of CA in our analysis is explore the main patterns and thereafter test them with Mantel tests. More specifically, as the row plot of dimensions 1 and 2 gives an impression of spatially structured variability, we sought to test it with a test of correlation (Mantel).

Another solution is reducing the variables included in the analysis, especially the ones that clearly differs from the Aurignacian attribution and coincide with sites that has reported stratigraphic

inconsistencies (e.g Chatelperronian points, MP points, LevalloisTech, DiscoidalTech, Microgravette). This will clean the data for the "noise" created by reporting lithic assemblage with possible artefact mixing or inconsistent contextual data.
The authors must conduct these steps to increase the reliability of the analysis and make their assumptions (Line 722-727 among others) more robust. I encourage them to dedicate time on this regard in order to build consistent quantitative analysis for the arrival of AMH into Iberia.

Thank you for this suggestion, it also partially overlaps with comments made by Reviewers 2 and 3. As mentioned above, we have trimmed the number of typological attributes to reduce potential noise in the CA and partial Mantel tests. These results are now given in the S2 File and in all cases they reflect those of the original analysis.

We should however note that we retained DiscoidalTech due its more frequent and temporally expansive occurrence. We have also added some commentary on the significance of discoidal technology in the Discussion of the Manuscript.

Discussion
Same mention for the Introduction section. I will avoid the term atypical or non-diagnostic from the discussions as they refer to what it should be included or not in an assemblage. But variability is part of the palaeolithic world, different populations organising technology in so many diverse ways according to factors such as duration, intensity, mobility, raw material availability, skills, culture. There are so many factors that can be behind the variability, but assemblages lacking chronocultural markers should not be defined as atypical, as there are not typical assemblages in palaeolithic archaeology. For example, the presence of several Chatelperronian points in small assemblages in Labeko Koba, Ekain or Cova Foradada have been interpreted as logistic hunting camps (Rios-Garaizar et al. 2012 and Morales et al. 2019). Chatelperronian points would make typical this assemblage in the Chatelperronian phylum? However, the limited number of artefacts make unable any information about average technological traits, reduction sequences, management of raw materials, refittings, spatial organisation, etc. Assemblages with no fossil-directors perhaps suggests that those ones are not that relevant to understand or study human behaviour and cultural phases among the Upper Palaeolithic record.
This is partially explained in 811-813, but those assemblages are incorporated to the data as part of the "variability" of the EUP in Iberia with any additional explanation or reflection about what do they mean with regards the general structure of the Aurignacian. The authors recognise this limitation (line 865-868)

We have removed the term 'atypical' as mentioned previously but retained the term 'undiagnostic'. This is because assemblages are commonly referred to as diagnostic/undiagnostic in the literature with reference to their cultural attribution. Moreover, one aspect of our study is to situate this observed variability within the Aquitaine model and so we believe these terms are useful.

We do of course agree that variability is the norm, rather than expectation (lines 185-187). However, we would argue that assemblages with no fossil directors are still relevant to understand or study human behaviour and cultural phases in the Upper Palaeolithic. In our view, such assemblages are able to expose underlying behavioural variation and the utility of existing cultural models to understand the past. To this end, we have rephrased the wording in lines 870-875 (previously lines 865-868).

Conclusions
Also, at the end the conclusions are not very well linked with the Introduction and some of the questions mentioned in that section. The authors might explore whether the spatial similarities

among assemblages are also consistent with the interpretative scenarios mentioned for dispersal of the AMH population in Iberia or previous models discussed such as the Ebro Frontier.

Thank you, we have now added some text that links the questions posed in the Introduction. Regarding the interpretative scenarios and models mentioned, we do not think that on this occasion our quantitative results provide robust answers to these topics.

Morales, J. I., A. Cebrià, A. Burguet-Coca, J. L. Fernández-Marchena, G. García-Argudo, A. Rodríguez-Hidalgo, M. Soto, S. Talamo, J.-M. Tejero, J. Vallverdú and J. M. Fullola (2019). "The Middle-to-Upper Paleolithic transition occupations from Cova Foradada (Calafell, NE Iberia)." PLOS ONE 14(5): e0215832. https://doi.org/10.1371/journal.pone.0215832

Rios-Garaizar, J., A. Arrizabalaga and A. Villaluenga (2012). "Haltes de chasse du Châtelperronien de la Péninsule Ibérique : Labeko Koba et Ekain (Pays Basque Péninsulaire)." L'Anthropologie 116(4): 532-549. https://doi.org/10.1016/j.anthro.2012.10.001

Minor comments
53-54 Repetition: novelty and innovations.

Thank you, amended via reworked introduction.

55 and 1019 tool-making groups is not a common term, use better “human groups”, “hunter gatherer groups”

Thank you, we have implemented this suggestion.

311 – Cova Gran is a EUP assemblage with no Middle Palaeolithic component.

This is a valid point. When we mention that Cova Gran level 497D has Middle Palaeolithic elements, we are referring to the strong signal of flake production (flakes comprise >80% of whole blanks) and the predominance of notches, denticulates and side-scrapers among the retouched tools (58% of the inventory), all of which gives the assemblage an ‘archaic aspect’ (Mora et al. 2018: 40). However, we accept that ‘Middle Palaeolithic’ might be inaccurate or misleading given that blade production is strongly represented, thank you.

711 I found this idea oversimplified. Of course there are networks among hunter-gatherers

We appreciate that the idea comes across oversimplified and have improved the wording of the relevant paragraph.

717 I suggest removing the idea of exchange as it is not proven and long-distance raw materials are a primary indicator of mobility.

As above, we are merely hypothesising and caveating that studies of networks are lacking. The long-distance transport of raw materials can also be the result of exchange. We note this recent study:

Sánchez de la Torre, M., et al. 2026. Far-reaching hunter-gatherer networks during the Last Glacial Maximum in Western Europe. *Science Advances*, 12(4), eadz7697. https://doi.org/10.1126/sciadv.adz7697.

758 Correction: The use of poor-quality chalcedony does not apply to the Magdalenian sequence of Cova Gran. There are recent works addressing this, also in Cova del Parco cave, also Magdalenian, that incorporates good quality evaporitic chert in the lithic assemblages. For some raw material constraints in the technological organisation, sites as La Viña, Pego do Diabo or Gato Preto reported industries mainly based on quartz.

Our understanding from the literature is that the same type of Garummian chalcedony is used in throughout the Upper Palaeolithic sequence of Cova Gran (Roy Sunyer, 2016; Martínez-Moreno et al. 2019) but did not hinder the production of blades and bladelets during the Magdalenian (Sánchez-Martínez et al., 2022). However, we accept this statement may be loosely supported so have removed it from the Manuscript.

Line 642 and Line 773 (among others) I will avoid the use of the term “atypical” or “non-diagnostic” of the discussion.

Thank you, we have addressed this in a previous response.

All in all, I believe this is a soundness contribution to the field that I hope the authors improve taking into account the suggestions here mentioned.

Thank you for taking the time to write this detailed and constructive review.

Reviewer #2: The paper by Canessa and de la Peña addresses one of the most intriguing regions in EUP research: the Iberian Peninsula. The archaeological record here seems to differ significantly from the broader models of change visible in France and Italy, particularly regarding the southern record. As the authors state, the variability in Iberia does not easily fit into these established European frameworks, a factor that contributed to J. Zilhão’s model of a very late Aurignacian presence in southern Iberia. While the picture is slowly changing thanks to new excavations and dating programs, certain issues remain open to debate (e.g., Bajondillo). In this context, this quantitative study is a welcome contribution.

First, I want to state that the goal of the authors is remarkable; this represents one of the few attempts to compare Aurignacian assemblages quantitatively across space and time. I truly appreciate the open sharing of datasets and the methodological workflow. The supplementary information is clear and well-structured, and I assume it will be published as a repository with a DOI based on the data statement. I was able to reproduce the main findings without issue using the well-written and organized R code. I also commend the additions to the Mantel tests to discuss spatial autocorrelation, as well as the use of an R package to calibrate dates and extract median ages. This aspect of the work is executed exceptionally well. However, despite these strengths, I have several concerns regarding the underlying data structure and methodology that need to be addressed before publication:

Thank you for your commendations of the study.

Presence/absence data: My main skepticism lies in the use of presence/absence data for testing variability across the Aurignacian. I question whether this approach is sufficiently high-resolution for the Upper Paleolithic, and specifically for a technocomplex like the Aurignacian, which is often defined by a set of features (e.g., carinated cores, Dufour bladelets) that are ubiquitous across its temporal span. The risk here is oversimplification, which may mask variability. Recent studies on

the Aurignacian demonstrate that it is often the frequency or relative importance of specific technological features, rather than their presence, that discriminates between different phases. By relying on presence/absence, the discussion risks becoming blurred. While Blinkhorn and Grove used a similar approach, they analyzed a much larger temporal and geographic span with MSA sites structured by raw materials and variables such as roughness. However, their scope and research questions were fundamentally different. I appreciate that the authors discuss these shortcomings in the discussion section, but I believe further validation is required

We fully understand the skepticism of using a presence-absence dataset to test variability across the Aurignacian. As we mention in the Manuscript, this approach results in a more coarse-grained resolution that some may find less satisfactory. However, we also explain in the Manuscript that the reporting of data is too inconsistent and variable to enable the analysis of continuous variables – like the frequency and proportion of artefact types – across such a large sample of assemblages. In other words, the analysis of continuous assemblage variables would shrink the sample of assemblages significantly, therefore reducing the spatial and temporal coverage of this study and defeating one of the study's main objectives. As we mention above in a response to Reviewer 1, it is common that older publications (of well known sites) report technological data in a partial and insufficient manner e.g., L'Arbreda level G and Cova Beneito levels B8–B9. Moreover, among some of the assemblages we included, the relevant publications do not even provide counts for basic artefact classes; instead, they are merely referenced as being present (e.g. blades and bladelets in L'Abreda level H or Aitzbitarte III levels Vb-c and Vb-u) or reported as coarse percentages (e.g. Bajondillo level 11). So, in order to test the variability across a large geographical and chronological span (i.e. the entire Iberian Peninsula and entire Aurignacian timeframe of 43-32 ka cal BP) and with the maximum possible number of assemblages, a presence-absence format was considered necessary

We would also like to add that our more inclusive and broad approach presents the first quantitative synthesis of techno-typological traits from the entire Iberian Aurignacian. In the coming year, we plan to build on these methods and results by analysing higher resolution datasets of Iberian assemblages. Despite the fact that such an analysis will reduce the sample of assemblages, it will nonetheless enable us to examine whether different patterns and findings emerge from a more fine-grained dataset. We have also now mentioned the possibility that the frequency and proportion of techno-typological features may generate a different result to the one we present in the Manuscript (lines 933-937).

Raw material variability: One way to strengthen the analysis is to test more directly if raw material availability affects the observed variability. Although I understand that data availability is limited, the strong spatial signal observed suggests that raw material constraints may play a significant role. Could the authors attempt to use broad groups of raw materials to test the outcomes? I suspect, for instance, that the presence of Quartz drives significant variability. While raw material cannot explain all variability, it likely plays a crucial role that should be explicitly tested, even if only at a coarse resolution.

We completely agree that this is an important variable that warrants testing. However, yet again, not all assemblages we analysed contain sufficient published data on raw material types and frequencies (for example, see publications for Cova Malladetes XII-XIVA, Cova Cendres level XIV-C, Cova Gran level 497D, El Castillo level 18C and La Viña XII-XIII(inf.)). Therefore, this selection criteria would: 1) eliminate highly interesting sites and assemblages and 2) prevent us from maximising the geographic and temporal scale of our analysis.

However, we wish to state again that our future study will incorporate frequencies of raw material types within multivariate analyses of continuous variables. Although this will restrict the spatio-temporal scope of the analysis, we agree that it will allow us to test more directly the extent

to which raw material is influencing variability and, therefore, our current suggestion that the spatial character of resources/behaviour is an important factor.

Regional sensitivity: I propose running an additional analysis focused exclusively on northern Iberia, where the data is more fine-grained and the dating is generally more reliable. Since the authors noted that northern assemblages appear more similar to one another, it would be valuable to see if the Mantel tests and partial Mantel tests yield different results when restricted to this region.

Thank you, we welcome this interesting suggestion. However, as our study is focused on a first-time quantitative synthesis of the whole Iberian Peninsula, we would prefer to postpone such an analysis for a future time. We feel that including it at this stage would expand the paper beyond it's primary scope and objectives, which is take a broad scale view of variability across the whole region.

Stratigraphic integrity and typological noise I argue that a presence/absence analysis must account for stratigraphic mixing and excavation errors, particularly when dealing with this specific set of sites. What happens if the analysis focuses on types clearly linked to the Aurignacian, excluding elements like Châtelperronian points? The inclusion of such types, or Levallois technology (which is not clearly documented in the Aurignacian), suggests that the analysis may be picking up on stratigraphic or post-depositional mixing rather than cultural variability. Looking at Figures 5 and 6, it appears that some of these "intrusive" blanks drive a large amount of variability in the CA analysis. Also, categories such as "core working" are not particularly useful in this context, as they are too broad to provide meaningful distinctions.

We agree that certain types (like Châtelperronain and Middle Palaeolithic points) may be the result of mixing but neither can we rule out behavioural explanations (e.g., convergent practices). Since we therefore cannot be certain that they are just noise, we originally chose to retain them in the analysis as a way of presenting the full spectrum of variability among Aurignacian assemblages. That being said, we have run a new analysis with these types excluded and integrated the results in the Manuscript and a new S2 File (see also Reviewer 1's comments above).

Regarding the use of the "CoreWorking" category, this amalgamates core tablets, core flanks, core rejuvenation flakes and crested/semi-crested pieces (as we now additionally show in the Sankey diagram in S1 Fig). We consider this category valid because they are technological practices that centre on the management and rejuvenation of cores and therefore offer information on the variability of technological practices documented in assemblages.

In conclusion, I truly appreciate the competent and mature discussion provided in the Manuscript. I believe this paper will serve as a starting point for expanding this specific research agenda. However, the authors need to more carefully address the limitations of presence/absence data and the necessity of collecting technological, quantitative data across sites to ensure the robustness of their conclusions.

Thank you for your evaluation of our work.

A minor comment: Why are only partial Mantel tests reported in the text and table? It would be helpful to include the full results.

Partial Mantel tests refer to Mantel tests that compare two independent matrices whilst controlling for the effect of a third (see first column of Table 2). As outlined in the Methods section, this is a

more robust procedure for accounting for autocorrelation than a simple Mantel test between two matrices.

However, when Moran's Spectral Randomisation is applied to remove the effect of spatial autocorrelation, it can only be applied to a 'simple' Mantel test (lines 494-496 and caption of Table 2). As simple Mantel test refers to a test between only two distance matrices.

Reviewer #3: We would like first to thank the editors for entrusting us with the peer review of this study. We should note, however, that our expertise does not encompass multivariate analysis, therefore we did not comment the specific methodological details of the statistical approach. This review is divided into three parts: an introductory section, a series of general suggestions, and finally detailed line-by-line comments.

The authors present a large-scale statistical analysis of lithic typo-technological data from Early Upper Palaeolitic (EUP) assemblages spanning about 10,000 years (43-32 ka cal BP) across the Iberian peninsula. The analysis aims to unravel the variability within and between the chrono-cultural technocomplexes characteristic of this period, namely the Proto-Aurignacian, Early Aurignacian, Evolved Aurignacian, and Late Aurignacian. Some assemblages lack diagnostic features typical of Aurignacian technocomplexes, raising the question of the validity and relevance of using "index fossils" to define archaeological cultures in this region and timeframe. To address this issue, the authors perform a combination of two multivariate analytical methods to test the correlation between inter-assemblage variability and spatial and temporal distances. Data were collected from the archaeological literature and operationalised as binary variables (presence/absence of traits), enabling the inclusion of an extensive number of assemblages (n=41). The dataset comprises both technological (n=13) and typological attributes (n=18).
The authors demonstrate that spatial distance significantly influences inter-assemblage lithic typo-technological variability. They highlight a marked divide between northern and southern assemblages, arguing that spatial proximity is a strong predictor of similarity. While several explanatory factors are considered (including information networks, foraging strategies, raw material availability, and taphonomic biases), the authors propose that inter-assemblage variability is mainly driven by resource distribution, and associated mobility patterns and social organisation. In parallel, they show that temporal distance does not affect inter-assemblage lithic typo-technological variability, thereby challenging the conventional chronological succession of the four Aurignacian phases as inferred from southwestern French archaeological sequences (the so-called "Aquitaine model"). The observed inconsistency of diagnostic Aurignacian features (e.g. carinated cores, Dufour bladelet sub-types, Aurignacian retouch), combined with the occurrence across technocomplexes of several undiagnostic traits (e.g. bipolar, discoid and ubiquitous flaking methods) lead the authors to questioning the spatio-temporal validity of these "index fossils." They ultimately advocate shifting the focus from the characterisation of archaeological cultures, potentially shaped by arbitrary research biases, towards a more process-oriented understanding of technological and behavioural change across this period.
In our view, this study offers an original and much-needed approach to the investigation of the EUP. The extensive spatial and temporal scope (the Iberian peninsula over more than 10,000 years), along with the substantial number of assemblages (n=41) and typo-technological attributes (n=31), are particularly noteworthy. This work represents a highly convincing effort of quantification of carefully selected qualitative data, and stands as an important contribution to cumulative, heuristic research, particularly through its commitment to open science principles. We would however like to suggest a few minor revisions, particularly in the discussion section.

We appreciate your detailed evaluation of our work, thank you.

General comments and suggestions

- Proportions of attributes:

The authors repeatedly acknowledge the limitations of simplifying lithic assemblage variables into binary presence/absence traits. We find their reasoning convincing and appreciate the transparency maintained throughout the paper. In particular, the argument that inconsistencies in the literature preclude the use of continuous variables (e.g. line 253) is well justified. However, we would suggest to further discuss the potential methodological biases arising from the lack of proportional representation of attributes. The binary system used in this study does not convey the relative importance of each attribute in relation with others. For instance, the proportion of core categories is often used to characterise technocomplexes. Excluding "generic core types" from the analysis (line 347) may therefore lead to an overestimation of the significance of diagnostic "index fossils" cores, such as carinated cores.

Thank you, we acknowledge the lack of commentary on the proportions of different artefact classes (see also our response to Reviewer 2 above). We consider this important and have added some text (lines 933-937).

We also appreciate that the exclusion of other core types results in a loss of information that may imply an elevated significance of other core types like carinated- or nosed-enscraper cores. This is a less than ideal scenario that we reluctantly had to accept due to the incomparability of core data. In other words, it would be equally problematic to include the full range of core classifications/terminologies found across publications (for example, PolyhedralCore, PrismaticCore, BladeCore, BladeletCore) because there will be overlap among some categories but not others. For instance, an assemblage reported to have PrismaticCores may appear different to an assemblage reported to have BladeCores and BladeletCores, but in reality those PrismaticCores may encompass cores for blades, bladelets or both blades and bladelets. As some publications do not provide details on the target products of PrismaticCores, there is no way to amalgamate or representatively capture the full spectrum of core classifications/terminologies without creating distortions of the underlying data.

- Technological attribute:

Recent studies have suggested that bladelet core management through the extraction of lateral and often asymmetrical blades represents a characteristic technological feature of the Proto-Aurignacian (Falcucci et al. 2017; Falcucci and Peresani 2018; Gennai et al. 2021; Gennai et al. 2025). We suggest considering the inclusion of a technological attribute related to lateral core management via the removal of "comma-like" blades (Falcucci et al 2020) to test the validity of this criterion for identifying Proto-Aurignacian assemblages in the Iberian peninsula. We believe that including this attribute would effectively complement the current list, which is relatively oriented towards lithic typology (18 typological versus 13 technological attributes). We would however understand that, once again, the variety of the literature used may refrain from integrating such a technological category of artefacts into the list of attributes.

We appreciate this suggestion. We wish this type of data — along with others we originally sought to include, like twisted bladelet blanks — was sufficiently found across the range of publications to be included in our analysis. Sadly, this is not the case and reflects the problems that underpin large-scale analyses of data that has been published across different decades.

However, we take these concerns in mind and have now reduced the typological attributes for CA and Mantel tests, also following the recommendations of Reviewers 1 and 2. These results are mentioned in the Manuscript and additionally in the new S2 File.

- Spatial representativity:

As said before, the large spatial scale of the study is greatly appreciated. Nevertheless, we would

like to raise a few concerns regarding the spatial representativity of the sample, which, in our opinion, warrants further discussion. Since the spatial segmentation of chaînes opératoires is known to vary across technocomplexes (e.g. Bordes et al. 2006 ; Porraz et al. 2010), the archaeological interpretation of the absence of diagnostic pieces (e.g. lines 909-912 and 956-957) may be affected by the incomplete excavation of occupations. This issue may be particularly pronounced at raw material procurement sites, where the range of activities is often very limited, and may be spatially segmented across broader territories. Furthermore, among the assemblages (n=41), only two sites are open-air, both attributed to the Evolved/Late Aurignacian. This raises the possibility that the presence or absence of variables, and consequently the potentially changing "functional solutions" observed across spatial and temporal scales (line 943), may depend heavily on the spatial (and thereof chronological) distribution of archaeological research itself. We believe this limitation has not been sufficiently emphasised in the current version of the Manuscript. Nevertheless, we recognise that the dataset derives from published sources and thus inevitably reflects diverse approaches and research traditions, which the authors have admirably synthesised, and standardised in a nuanced manner.

We wholeheartedly agree that the absence of diagnostic elements can be influenced by the spatial extent of excavation, as every excavation is itself a spatial sample. It also true that the composition of the two open-air sites may be shaped by how those sites were used. In order to bring the these points into greater attention, we have added some additional commentary to the Discussion section which and provided information on the spatial extent of Cova Gran level 497D ($55m^2$) to make our argument stronger.

- Bipolar and discoid methods:

We find the discussion of undiagnostic attributes across spatial and temporal dimensions (lines 923-924) particularly engaging. However, while the authors address generic flaking methods, they only briefly mention bipolar and discoid methods, which, from our perspective, appear far less ubiquitous during the Upper Palaeolithic than generic flake production, and therefore potentially more informative. In particular, are bipolar and discoid methods used exclusively to produce flakes, or are they also integrated into laminar reduction sequences? We would encourage the authors to elaborate further on the significance and distribution of bipolar and discoid methods across EUP assemblages.

Thank you for the suggestion, we have elaborated on the significance of bipolar and discoidal technology in lines 1001-1008. Unfortunately, details on how bipolar knapping was integrated into knapping schemes is not always specified or determinable, so we could not integrate it into the discussion. Having said that, Zilhão et al. 2017 comment that the bipolar cores from OH16 and OH17 from La Boja indicate a strategy to extract as much cutting edge as possible from a given volume of material. In this case, they could represent the last stage of reduction of prismatic nodules first knapped by freehand percussion.

- Cross-regional perspective:

The Aquitaine model is primarily grounded in qualitative technological analyses of long archaeological sequences in southwestern France. To our knowledge, no large-scale quantitative approach comparable to the one proposed here has yet been undertaken in this region. We consider this absence a significant limitation for meaningful cumulative comparisons between southwestern France and the Iberian peninsula, thereby hindering any conclusive attempts to evaluate or challenge the Aquitaine model. Given that large-scale quantitative studies remain scarce, we recommend integrating in the Manuscript what appears to be the only other comparable and recent effort to assess technological similarity among EUP assemblages, namely Gennai et al. 2025 (https://doi.org/10.1371/journal.pone.0331393). Although based on a smaller

set of assemblages, spanning a narrower temporal range but a broader spatial scale, this dataset was prepared for multivariate analysis and is openly available, allowing it to be processed according to the methodology employed in the present study and directly compared with the Iberian results.
Looking ahead, we enthusiastically encourage the authors to broaden even more the spatial coverage of their analysis, potentially through collaboration with teams engaged in similar quantitative research. We strongly believe that fostering convergence among research groups working in different geographical areas under open science standards will be a crucial step towards a deeper understanding of the processes of technological and cultural change during the EUP.

We appreciate this suggestion but feel that integrating Gennai et al. 2025 into the Manuscript would push the paper beyond its intended scope – that being a study on the techno-typological variability of assemblages specific to Iberia . We agree that the Aquitaine model is mainly typological in nature but would emphasise that our evaluation of it is made on the basis that we are able to: 1) show heterogeneity within and between temporal classes of assemblages, thereby challenging the fundamental diachronic element of the model and 2) prove that spatial distance predicts inter-assemblage dissimilarities, meaning that geographically-specific models (like the Aquitaine) may not, as a general rule, apply to other regions (and not just Iberia, for that matter).

We are also in total agreement with broadening the geographical scope of our analysis at a future stage. The concept of this study was generated within the PhD project of the first author and it is currently intended that additional works will be carried out that build on the data/analyses so far collected/conducted. We also welcome the potential to collaborate with researchers engaging in similar topics and hope that our publication will help make this happen.

- Aurignacian expressions:
We greatly appreciate the broader interpretations derived from the empirical results, particularly the proposition at lines 955-959 that inconsistent proportions of diagnostic and undiagnostic features in assemblages may alternatively reflect either a diverse manifestation of a generic and widely shared Aurignacian technological system, or differing strengths of ties between groups within this spatial and temporal frame, as expressed by the scattered emergence of "low-fidelity innovations." Yet, whether these should be termed "innovations" is debatable, see for instance the introduction in O'Brien and Shennan (ed) for different definitions of the term.

Thank you for directing us to this literature – we appreciate that "innovations" might not be the best term. We have hence replaced the word in all relevant areas of the Manuscript.

Line-by-line comments and suggestions
- 72: "discreet" → "discrete"

Thank you for spotting that.

- 96: add Gennai et al. 2025, where they assess the technological variability across EUP sites

We appreciate this suggestion, thank you.

- 150: add Djakovic et al. 2022, where they discuss the timing of overlap between CP and PA

Thank you, we have added it to refer the reader to this complimentary study.

- 341: “Carinated.EndsraperCores” → “Carinated.EndscraperCores”

Thank you, amended.

- 404-405: add Le Brun-Ricalens et al. 2009, where they discuss the typological problem of Krems versus Font-Yves points

Thank you for this good suggestion, we have added the publication.

- 417-418: “piéces esquilles” → “pièces esquillées”

Corrected, thanks.

- 463: “Within in each” → “Within each”

Amended via deleted sentences (Reviewer 1 comment on shortening methods).

- 509: “Aurignacian blades, which are only known from northern sites”: does this statement only targets the Iberian peninsula or is it broader?

This statement refers to the Iberian Peninsula which we now have now clarified.

- 685-686: add Le Brun-Ricalens et al. 2009; Tsanova et al. 2012

Thank you, we agree that Le Brun-Ricalens et al. 2009 is a relevant publication and have added it. However, we have read the publication by Tsanova et al. 2012 carefully and are inclined to say that although the authors highlight regional variability (mainly typological) among Early Upper Palaeolithic assemblages, they do not make an explicit argument that the Aurignacian is a spatially and temporally variable phenomenon demonstrating an inconsistent representation of behaviours.

- 744 and 746: numbers “3” and “2” should be written in letters
- 791: “of with 268” → “of which 268”
- 877: “Rècent” → “Récent”
- 1044: “phdthesis” → “PhD thesis”
- 1059, 1062, 1188: “Arrizabalaga, À.”: accent on À not appearing at lines 1050 and 1054
- 1178: “paléolithique” → “Paléolithique”
- 1267: “paleolithique superieur” → “Paléolithique supérieur”
- 1645: “tronquèes” → “tronquées”
- 1646: “de France” → “française”
- 1683: “Península” → “Péninsule”
- 1683-1884: “Paleolithique moyen recent et paleolithique superieur” → “Paléolithique moyen récent et Paléolithique supérieur”

Many thanks, we have corrected all the above.

- 1746-1747: remove squares

The squares are probably an error arising from pdf conversion as they not appear in the .docx version of the Manuscript file. We will take note of this and inform the editorial team if the problem persists.

- 1757: “contain” to remove

Thank you, we have clarified the caption.

References used in the review

Bordes, J.-G., 2006. ‘News from the West: a reevaluation of the classical Aurignacian sequences of the Périgord.’ In: Bar-Yosef, O., Zilhão, J. (Eds.). Towards a Definition of the Aurignacian. Lisboa: Instituto Português de Arqueologia, 147–171.

Djakovic, I., Key, A., Soressi, M, 2022. ‘Optimal linear estimation models predict 1400–2900 years of overlap between Homo Sapiens and Neandertals prior to their Disappearance from France and Northern Spain.’ Scientific Reports 12 (1): 15000. https://doi.org/10.1038/s41598-022-19162-z.

Falcucci, A., Conard, N. J., Peresani, M., 2017. ‘A critical assessment of the Protoaurignacian lithic technology at Fumane Cave and its implications for the definition of the earliest Aurignacian.’ PLOS ONE, 12(12), e0189241. https://doi.org/10.1371/journal.pone.0189241.

Falcucci, A., Conard, N. J., Peresani, M., 2020. ‘Breaking through the Aquitaine frame: A re-evaluation on the significance of regional variants during the Aurignacian as seen from a key record in southern Europe.’ Journal of anthropological sciences = Rivista di antropologia: JASS, 98, 99–140. https://doi.org/10.4436/JASS.98021.

Falcucci, A., Peresani, M., 2018. ‘Protoaurignacian core reduction procedures: blade and bladelet technologies at Fumane Cave’. Lithic Technology 43 (2): 125–40. https://doi.org/10.1080/01977261.2018.1439681.

Gennai, J., Falcucci, A., Niochet, V., Peresani, M., Richter, J., Soressi, M., 2025. ‘Tracking the emergence of the Upper Palaeolithic in Western Asia and Europe: a multiple correspondence analysis of Protoaurignacian and Southern Ahmarian lithics.’ PLOS ONE 20 (9): e0331393. https://doi.org/10.1371/journal.pone.0331393.

Gennai, J., Peresani, M., Richter, J., 2021. ‘Blades, bladelets or blade(let)s? Investigating early upper Palaeolithic technology and taxonomical considerations.’ Quartär 68: 71-116.

Le Brun-Ricalens, F., Bordes, J.-G., Eizenberg, L., 2009. ‘A crossed-glance between southern European and Middle-Near Eastern early Upper Palaeolithic lithic technocomplexes. Existing models, new perspectives.’ In: Camps, M., Szmidt, C. C., eds. The Mediterranean from 50 000 to 25 000 BP: Turning Points and New Directions. Oxford: Oxbow Books, 11–33.

O’Brien, M. J., Shennan, S. (Ed.), 2010. Innovation in Cultural Systems: Contributions from Evolutionary Anthropology. Vienna Series in Theoretical Biology, MIT Press.

Porraz, G., Simon, P., Pasquini, A., 2010. ‘Identité technique et comportements économiques des groupes proto-aurignaciens à la grotte de l’Observatoire (principauté de Monaco)’. Gallia préhistoire 52 (1): 33–59. https://doi.org/10.3406/galip.2010.2470.

Tsanova, T., Zwyns, N., Eizenberg, L., Teyssandier, N., Le Brun-Ricalens, F., Otte, M., 2012. ‘Le plus petit dénominateur commun : réflexion sur la variabilité des ensembles lamellaires du Paléolithique supérieur ancien d’Eurasie. Un bilan autour des exemples de Kozarnika (Est des Balkans) et Yafteh (Zagros central).’ L’Anthropologie 116 (4): 469–509. https://doi.org/10.1016/j.anthro.2011.10.005.
